# Evolution and Global Transmission of a Multidrug-Resistant, Community-Associated Methicillin-Resistant Staphylococcus aureus Lineage from the Indian Subcontinent

**DOI:** 10.1128/mBio.01105-19

**Published:** 2019-11-26

**Authors:** Eike J. Steinig, Sebastian Duchene, D. Ashley Robinson, Stefan Monecke, Maho Yokoyama, Maisem Laabei, Peter Slickers, Patiyan Andersson, Deborah Williamson, Angela Kearns, Richard V. Goering, Elizabeth Dickson, Ralf Ehricht, Margaret Ip, Matthew V. N. O’Sullivan, Geoffrey W. Coombs, Andreas Petersen, Grainne Brennan, Anna C. Shore, David C. Coleman, Annalisa Pantosti, Herminia de Lencastre, Henrik Westh, Nobumichi Kobayashi, Helen Heffernan, Birgit Strommenger, Franziska Layer, Stefan Weber, Hege Vangstein Aamot, Leila Skakni, Sharon J. Peacock, Derek Sarovich, Simon Harris, Julian Parkhill, Ruth C. Massey, Mathew T. G. Holden, Stephen D. Bentley, Steven Y. C. Tong

**Affiliations:** a Menzies School of Health Research, Darwin, Australia; b Australian Institute of Tropical Health and Medicine, Townsville, Australia; c Department of Microbiology and Immunology, University of Melbourne at The Peter Doherty Institute for Infection and Immunity, Melbourne, Australia; d University of Mississippi Medical Center, Jackson, Mississippi, USA; e Leibniz Institute of Photonic Technology (IPHT), Jena, Germany; f InfectoGnostics Research Campus, Jena, Germany; g Technical University of Dresden, Dresden, Germany; h Department of Biology and Biochemistry, University of Bath, Bath, United Kingdom; i Doherty Applied Microbial Genomics, Department of Microbiology & Immunology, The University of Melbourne at The Peter Doherty Institute for Infection and Immunity, Melbourne, Australia; j Microbiological Diagnostic Unit Public Health Laboratory, Department of Microbiology & Immunology, The University of Melbourne at The Peter Doherty Institute for Infection and Immunity, Melbourne, Australia; k Public Health England, National Infection Service, London, United Kingdom; l Creighton University, Omaha, Nebraska, USA; m Scottish Microbiology Reference Laboratories, Glasgow, United Kingdom; n The Chinese University of Hong Kong, Hong Kong; o Marie Bashir Institute for Infectious Diseases and Biosecurity, University of Sydney, Sydney, Australia, and New Wales Health Pathology, Westmead Hospital, Sydney, Australia; p School of Veterinary and Laboratory Sciences, Murdoch University, Murdoch, Australia; q Statens Serum Institut, Copenhagen, Denmark; r National MRSA Reference Laboratory, St. James’s Hospital, Dublin, Ireland; s Microbiology Research Unit, School of Dental Science, University of Dublin, Trinity College Dublin, Dublin, Ireland; t Istituto Superiore di Sanità, Rome, Italy; u Instituto de Tecnologia Química e Biológica, Oeiras, Portugal; v The Rockefeller University, New York, New York, USA; w University of Copenhagen, Copenhagen, Denmark; x Hvidovre University Hospital, Hvidovre, Denmark; y Sapporo Medical University, Sapporo, Japan; z Institute of Environmental Science and Research, Wellington, New Zealand; aa Robert Koch Institute, Wernigerode, Germany; bb Sheikh Khalifa Medical City, Abu Dhabi, United Arab Emirates; cc Akershus University Hospital, Lørenskog, Norway; dd King Fahd Medical City, Riyadh, Kingdom of Saudi Arabia; ee London School of Hygiene and Tropical Medicine, London, United Kingdom; ff Sunshine Coast University, Sippy Downs, Australia; gg Wellcome Sanger Institute, Cambridge, United Kingdom; hh School of Cellular and Molecular Medicine, University of Bristol, Bristol, United Kingdom; ii University of St. Andrews, St. Andrews, United Kingdom; jj Victorian Infectious Disease Service, The Royal Melbourne Hospital, and Doherty Department, University of Melbourne, Peter Doherty Institute for Infection and Immunity, Victoria, Australia; kk Department of Veterinary Medicine, University of Cambridge, Cambridge, United Kingdom; CHOP; New York University School of Medicine

**Keywords:** antimicrobial resistance, Bengal Bay, CA-MRSA, genomic epidemiology, global transmission, India, phenotyping, phylodynamics, ST772, South Asia, *Staphylococcus aureus*, WGS

## Abstract

The evolution and global transmission of antimicrobial resistance have been well documented for Gram-negative bacteria and health care-associated epidemic pathogens, often emerging from regions with heavy antimicrobial use. However, the degree to which similar processes occur with Gram-positive bacteria in the community setting is less well understood. In this study, we traced the recent origins and global spread of a multidrug-resistant, community-associated Staphylococcus aureus lineage from the Indian subcontinent, the Bengal Bay clone (ST772). We generated whole-genome sequence data of 340 isolates from 14 countries, including the first isolates from Bangladesh and India, to reconstruct the evolutionary history and genomic epidemiology of the lineage. Our data show that the clone emerged on the Indian subcontinent in the early 1960s and disseminated rapidly in the 1990s. Short-term outbreaks in community and health care settings occurred following intercontinental transmission, typically associated with travel and family contacts on the subcontinent, but ongoing endemic transmission was uncommon. Acquisition of a multidrug resistance integrated plasmid was instrumental in the emergence of a single dominant and globally disseminated clade in the early 1990s. Phenotypic data on biofilm, growth, and toxicity point to antimicrobial resistance as the driving force in the evolution of ST772. The Bengal Bay clone therefore combines the multidrug resistance of traditional health care-associated clones with the epidemiological transmission of community-associated methicillin-resistant S. aureus (MRSA). Our study demonstrates the importance of whole-genome sequencing for tracking the evolution of emerging and resistant pathogens. It provides a critical framework for ongoing surveillance of the clone on the Indian subcontinent and elsewhere.

## Abstract (precis)

The Bengal Bay clone (ST772) is a community-associated and multidrug-resistant Staphylococcus aureus lineage first isolated from Bangladesh and India in 2004. In this study, we showed that the Bengal Bay clone emerged from a virulent progenitor circulating on the Indian subcontinent. Its subsequent global transmission was associated with travel or family contact in the region. ST772 progressively acquired specific resistance elements at limited cost to its fitness and continues to be exported globally, resulting in small-scale community and health care outbreaks. The Bengal Bay clone therefore combines the virulence potential and epidemiology of community-associated clones with the multidrug resistance of health care-associated S. aureus lineages. This study demonstrates the importance of whole-genome sequencing for the surveillance of highly antibiotic-resistant pathogens, which may emerge in the community setting of regions with poor antibiotic stewardship and rapidly spread into hospitals and communities across the world.

## INTRODUCTION

Methicillin-resistant Staphylococcus aureus (MRSA) is a major human pathogen with a propensity to develop antibiotic resistance, complicating treatment and allowing persistence in environments where there is antibiotic selection pressure. While multidrug resistance has traditionally been the domain of health care-associated strains, the emergence of strains in the community setting that are also resistant to multiple antibiotics poses a significant challenge to infection control and public health (1). Given the heavy burden and costs associated with MRSA infections (2, 3), there is an urgent need to elucidate the patterns and drivers behind the emergence of drug-resistant community-associated MRSA lineages.

Over the last few years, several population genomic studies have started to unravel the evolutionary history of community-associated S. aureus lineages emerging in specific regions of the world. The prototype of these clones is the diverse USA300 lineage (sequence type 8 [ST8]), forming distinct genetic lineages in North America and South America (4, 5), including distinct clades in Europe and Africa (6). The East Asia clone (ST59) has diverged into two distinct lineages, with evidence of establishment in Taiwan and North America (7), while the ST80 lineage originated in North Africa but went through a notable population expansion to become the dominant community-associated lineage in North Africa, the Middle East, and Europe (8). On the Australian continent, ST93 emerged in indigenous communities of Western Australia and the Northern Territory and spread to the eastern seaboard and sporadically overseas, while also forming a clade associated with Pacific Islander and Maori populations in New Zealand (9).

This diversity of regional evolutionary histories is reflected in the various factors that have been suggested to contribute to the emergence and establishment of community-associated clones. For instance, acquisition of Panton-Valentine leucocidin (PVL), a mutation in the capsule gene *cap5D*, and acquisition of the SCC*mec-*IV and ACME/COMER elements played a defining role in the regional evolution of USA300 (6). While acquisition of a typical SCC*mec*-IV element was also associated with the emergence of the eastern seaboard clade of ST93, host population factors such as household poverty, crowding, and a high burden of skin infections have been postulated to be associated with both the emergence in indigenous populations of Australia and the establishment of a Pacific Islander-associated clade in New Zealand (9). In contrast, the population expansion of the ST80 lineage was linked to the acquisition of a SCC*mec*-IV element and fusidic acid resistance, and enrichment of resistance determinants in the Taiwan clade of ST59 suggests a strong contribution for its emergence and persistence in East Asia. While there is some evidence from these studies that resistance acquisition can be a driving force behind the regional emergence of community-associated MRSA clones, there is a lack of data on strains from critical regions that are considered hot spots for the emergence of multidrug-resistant pathogens, such as the Indian subcontinent.

In 2004, a novel S. aureus clone designated ST772 was isolated from hospitals in Bangladesh (10) and from a community setting study in India (11), where it continued to be reported in community- and health care-associated environments (see data posted at https://doi.org/10.6084/m9.figshare.8061887.v3) (12). Similar to other S. aureus strains, ST772 primarily causes skin and soft tissue infections, but more severe manifestations, such as bacteremia and necrotizing pneumonia, have been observed. Its potential for infiltration into nosocomial environments (13–16) and resistance to multiple classes of commonly used antibiotics (including aminoglycosides, β-lactams, fluoroquinolones, macrolides, and trimethoprim) (16–18) have made ST772 an alarming public health concern on the Indian subcontinent and elsewhere. Over the last decade, the clone has been isolated from community and hospital environments in Asia, Australasia, Africa, the Middle East, and Europe (see data posted at https://doi.org/10.6084/m9.figshare.8061887.v3). As a consequence of its discovery, distribution, and epidemiology, the lineage has been informally dubbed the Bengal Bay clone (19). Despite clinical and epidemiological hints for a recent and widespread dissemination of ST772, a unified perspective on the global evolutionary history and emergence of the clone is lacking.

In this study, we analyzed whole-genome sequences from a globally representative collection of 340 ST772 strains to elucidate the key events associated with the emergence and global spread of a multidrug-resistant community-associated MRSA clone. Our analysis suggests that the clone originated on the Indian subcontinent in the 1960s and rapidly expanded through the 1990s and early 2000s. We found that international travel and family connections to the region (India, Bangladesh, Nepal, and Pakistan) were closely linked with the global spread of the lineage. Genome integration of a multidrug resistance plasmid appeared to be a driver in the emergence of a dominant clade (ST772-A) in the early 1990s.

## RESULTS

**FIG 1:**
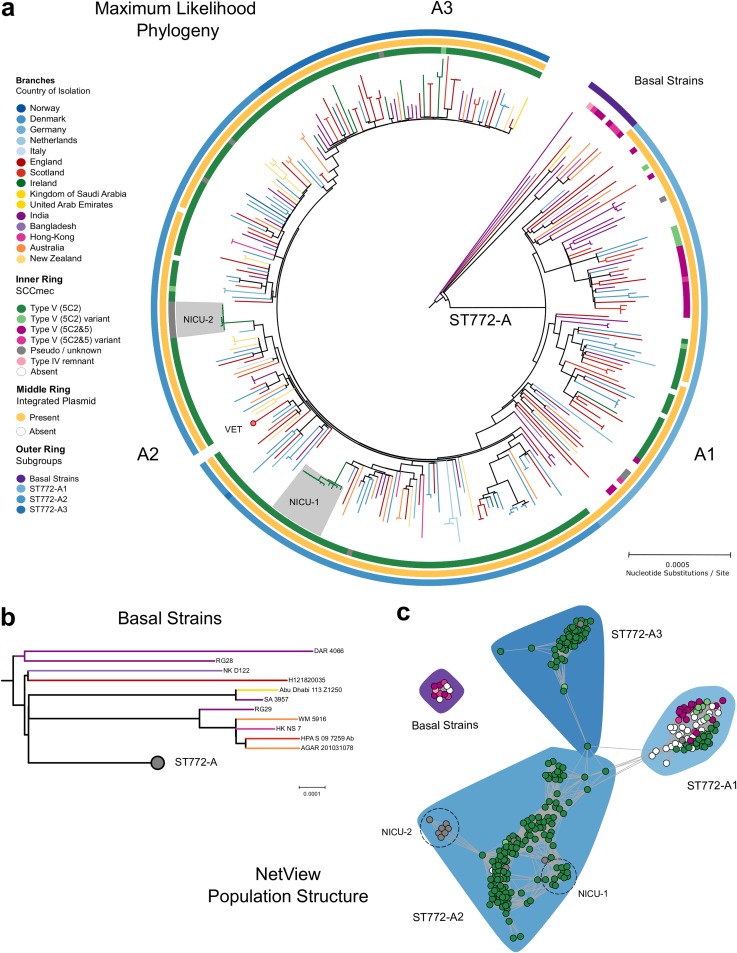
Evolutionary history and population structure of ST772. (a) Maximum likelihood phylogeny of ST772 (*n* = 340) based on 7,063 core genome SNPs. Branch colors indicate country of isolation, the inner ring delineates presence and type of SCC*mec*, the middle ring shows presence of the integrated resistance plasmid, and the outer ring indicates community membership of the population graph shown in panel c. Communities match the tree topology, with several basal isolates (*n* = 11) and a single derived clade, ST772-A (*n* = 329), composed of three population subgroups (A1 to A3). Isolates from two outbreaks in neonatal intensive care units in Ireland are indicated in gray (NICU-1 and NICU-2). Only one representative isolate from longitudinal sampling of a single health care worker (VET, *n* = 39) is shown (red circle). (b) Basal strains of ST772 showing positions of isolates from India and Bangladesh at the root of the phylogeny (RG28, DAR4066, and NKD122). (c) Population graph based on pairwise SNP distances, showing SCC*mec* type (node color as for panel a) and population subgroups (polygons, A1 to A3). Dashed circles indicate hospital-associated outbreaks in Ireland (NICU-1 and NICU-2).

We generated whole-genome sequence data for 354 S. aureus ST772 isolates collected across Australasia, South Asia, Hong Kong, the Middle East, and Europe between 2004 and 2013 (see data posted at https://doi.org/10.6084/m9.figshare.8061887.v3). Fourteen isolates were excluded after initial quality control due to contamination, and the remainder mapped with 165× average coverage against the PacBio reference genome DAR4145 (18) from Mumbai. From a core genome of 2,545,215 bp, there were 7,063 single nucleotide polymorphisms (SNPs). Phylogenetic analysis using core genome SNPs revealed little geographic structure within the lineage (Fig. 1a). Eleven ST772 methicillin-susceptible S. aureus (MSSA) and MRSA strains were basal to a single globally distributed clade (ST772-A; *n* = 329) that harbored an integrated resistance plasmid (IRP) described for the reference genome DAR4145 (18) (Fig. 1a and b). Population network analysis distinguished three distinct subgroups within ST772-A (Fig. 1a and c): an early-branching subgroup harboring multiple subtypes of the staphylococcal chromosome cassette (SCC*mec-*V) (A1; *n* = 81), a dominant subgroup (A2; *n* = 153), and an emerging subgroup (A3; *n* = 56); the last two exclusively harbor a short variant of SCC*mec*-V.

### Emergence and global spread from the Indian subcontinent.

**FIG 2:**
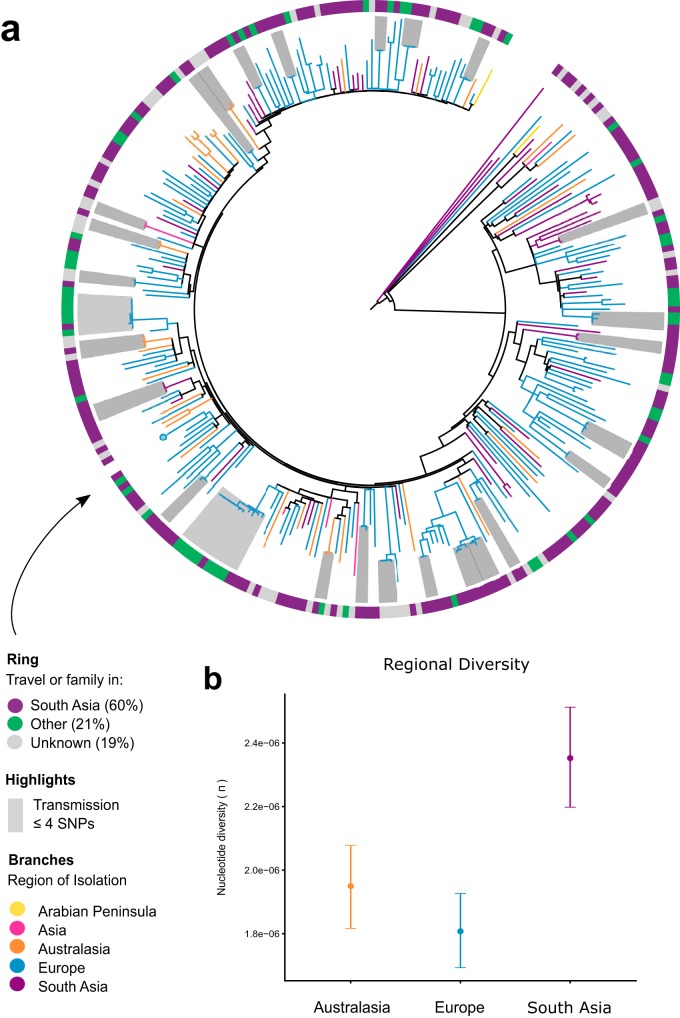
Molecular epidemiology of ST772. (a) Patient family or travel background in South Asia (India, Pakistan, Nepal, and Bangladesh) (59.5%, purple), or other countries (21.2%, green) or unknown status (19.3%, gray) is widely distributed across the phylogenetic topology of ST772 (*n* = 340). Only one representative isolate from longitudinal sampling of a single health care worker (*n* = 39) is included (circle). Clusters of isolates that are within 4 SNPs of each other and thus likely instances of transmission are shaded gray. (b) Average pairwise nucleotide diversity per site (π), measured by region (Australasia: orange, *n* = 36; Europe: blue, *n* = 244; South Asia: purple, *n* = 52). Error bars indicate 95% confidence intervals using nonparametric bootstrapping. Isolates from the Arabian Peninsula (*n* = 2) and Hong Kong (*n* = 6) were excluded from the diversity analysis due to the small number of samples from these regions.

Epidemiological and genomic characteristics of ST772 were consistent with an evolutionary origin from the Indian subcontinent. Sixty percent of isolates in this study were collected from patients with family or travel background in Bangladesh, India, Nepal, or Pakistan; the other isolates had unknown origins (19%) or were from other countries (21%) (Fig. 2a). We found more isolates from India and Bangladesh among the basal strains than clade ST772-A (Fisher’s exact test, 5/11 versus 47/291, *P* = 0.026), suggesting an association of the emergence of the clone with the Indian subcontinent. In particular, three isolates from India and Bangladesh were basal in the (outgroup-rooted) maximum likelihood (ML) phylogeny (Fig. 1b and Fig. S1A), including two MSSA samples from the original isolations in 2004 (RG28 and NKD122). Isolates recovered from South Asia were genetically more diverse than isolates from Australasia and Europe, supporting an origin from the Indian subcontinent (Fig. 2b and Fig. S1B).

10.1128/mBio.01105-19.1 FIG S1 (A) Maximum likelihood (GTR + Γ) phylogeny based on 25,701 SNPs rooted on outgroup strain MW2 (CC1, SRR592258) and including additional strains from another single-locus variant of CC 1, ST573 (*n* = 10), with branch lengths (a) and as a cladogram with 100 bootstrap support values (b). The phylogeny resolves the basal strain topology and is highly similar to the Least Squares Dating (LSD)-estimated root and the midpoint-rooted within-lineage phylogeny of ST772 (Fig. 1a and b), with strains from India at the base of ST772 (DAR4066 and RG28; bootstrap support for basal position outside remainder of strains, 100%). (B) Distribution of pairwise SNP distances between isolates from regions where *n* > 10 (orange: Australasia, blue: Europe, purple: South Asia) showing histogram representation with a distinction between the dominant clade ST772-A (distribution on the left) and basal strains (distribution on the right) (a) and box plot of pairwise SNP distance for each region (b). Kruskal-Wallis test on pairwise SNP distances suggests significant differences between the regions (χ^2^ = 171.22, df = 2, and *P* < 1 × 10^−6^). *Post hoc* Dunn’s test with Bonferroni correction for pairwise multiple comparisons demonstrates significant differences between all pairwise combinations (*P* < 1 × 10^−6^). (C) Distribution of methicillin-susceptible ST772 (MSSA). (a) Density distribution of patristic distance (in years using LSD-rooted maximum likelihood phylogeny) to the root of the phylogeny for MSSA and MRSA, demonstrating basal positions of MSSA isolates in the LSD-rooted and dated phylogeny of ST772. (b) Proportion of MSSA compared to MRSA isolates within regions. South Asia (*n* = 52) shows the highest proportion of MSSA isolates in concordance with a hypothesized MSSA progenitor of ST772 on the Indian subcontinent. (D) Regression analysis and date randomizations for LSD of ML phylogenies computed from core variants with PhyML under the GTR + Γ model after removing recombination (Gubbins, 6,907 SNPs). Each blue point in the regression plots corresponds to the distance from the root to a tip in the tree, and the solid blue line is the least-squares regression. In the date randomizations, the red line indicates the substitution rate estimate from LSD using the correct sampling times. The gray bars correspond to the histogram of substitution rate estimates obtained by randomizing the sampling times 100 times, such that they represent the null distribution of rate estimates under no temporal structure. The rate estimate with the correct sampling times is not within the range of those obtained using randomizations, indicating that the data have strong temporal structure. Download FIG S1, PDF file, 0.3 MB. Copyright © 2019 Steinig et al. 2019 Steinig et al. This content is distributed under the terms of the Creative Commons Attribution 4.0 International license.

Consistent with a methicillin-susceptible progenitor, a higher proportion of MSSA isolates was found among the basal isolates (Fisher’s exact test, 4/11 versus 31/291, *P* = 0.028), and MSSA isolates demonstrated a lower patristic distance to the root of the maximum likelihood phylogeny than MRSA (Fig. S1C). Although it appears that MSSA is proportionately more common in South Asia, it is also possible that the observed distribution may be related to nonstructured sampling (Fig. S1C).

**FIG 3:**
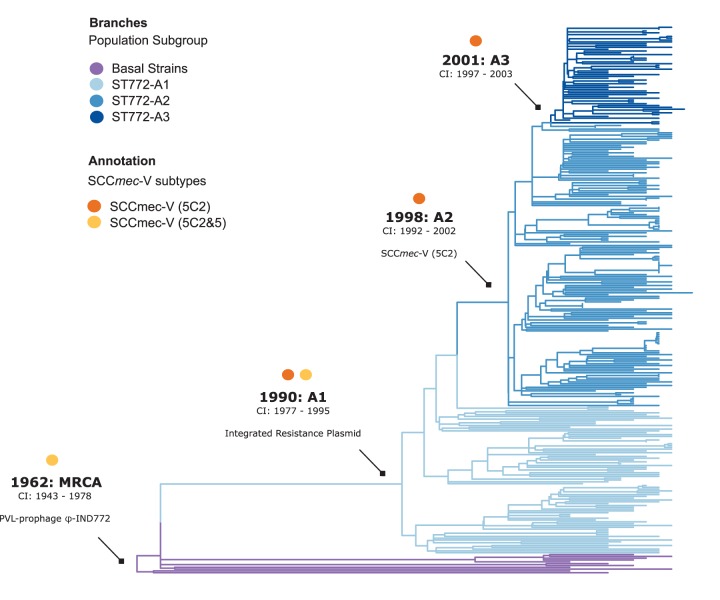
Molecular-clock estimates in the emergence of ST772. Shown is a phylogenetic time tree with the timescale estimated in Least Squares Dating (LSD). The annotations for nodes represent the time of origin (in years) of basal strains and subgroups A1, A2, A3, including 95% confidence intervals (CIs) and the most recent common ancestor (MRCA) of ST772. Tips are colored according to the subgroup, and the position of the root was optimized during the analysis. Arrows indicate acquisition of three critical mobile genetic elements: the PVL/*sea*-prophage φ-IND772, an integrated multidrug resistance plasmid, and the short staphylococcal cassette chromosome SCC*mec-*V (5C2).

Phylogenetic dating suggests an initial divergence of the ancestral ST772 population in 1962 (age of root node, 1961.94; confidence interval [CI], 1942.75 to 1977.69), with a core genome substitution rate of 1.18 × 10^−6^ substitutions/site/year after removing recombination (Fig. 3 and Fig. S1D). This was followed by the emergence of the dominant clade ST772-A and its population subgroups in the early 1990s (ST772-A divergence, 1990.02; 95% CI, 1977.5 to 1995.27). The geographic pattern of dissemination is heterogeneous (Fig. 1a). There was no evidence of widespread endemic dissemination of the clone following intercontinental transmission, although localized health care-associated outbreak clusters occurred in neonatal intensive care units (NICUs) in Ireland (NICU-1 and NICU-2 [Fig. 1a and Fig. S2]) (20) and have been reported from other countries in Europe (16) and South Asia (13–15). While some localized spread in the community was observed among our isolates, patients in local transmission clusters often had traveled to or had family in South Asia (19/27 clusters [Fig. S2]).

10.1128/mBio.01105-19.2 FIG S2 Transmission clusters in ST772 (see also data posted at https://doi.org/10.6084/m9.figshare.8061887.v3). Shown are connected components of an undirected graph with a threshold on pairwise SNP distance of 4 SNPs and mapped to maximum likelihood phylogeny of ST772. Inner and outer rings depict family and travel links to South Asia (India, Pakistan, and Nepal), while node colors within the dotted circles of clusters depict combined epidemiological link to South Asia (see Materials and Methods). Transmission clusters detected with network approach are in gray within the phylogeny of ST772. Most clusters (19/27) indicate a family or travel link to South Asia. Some of these cluster may indicate transmission within households, for instance, cluster 6 (husband and wife who both travelled to India) or cluster 7 and cluster 8, which include putative transmission between family members who also have family links to South Asia. Other clusters (cluster 2, 12, 23, and 26) have at least one member with links to South Asia and another without links to the Indian subcontinent, indicating spread within the community. NICU-1 and NICU-2 represent outbreaks in neonate intensive care units in Ireland. In NICU-1, the suspected index case was a staff member (M11-0092) who had been hospitalized in India, where she had traveled shortly before the recovery of the first isolate and where she had given birth to her child (M11-0167). The child was screened after detection in the index staff member, following the first three neonate cases in 2010 (isolates M10). Download FIG S2, PDF file, 0.2 MB. Copyright © 2019 Steinig et al. 2019 Steinig et al. This content is distributed under the terms of the Creative Commons Attribution 4.0 International license.

### Antibiotic resistance acquisition is associated with emergence and dissemination.

We examined the distribution of virulence factors, antibiotic resistance determinants, and mutations in coding regions to identify the genomic drivers in the emergence and dissemination of ST772. Nearly all isolates (336/340) carried the Panton-Valentine leucocidin (PVL) genes *lukS/F*, most isolates (326/340) carried the associated enterotoxin A gene (*sea*), and all isolates carried *scn*. This indicates a nearly universal carriage, across all clades, of both a truncated *hlb*-converting prophage (the typically associated staphylokinase gene *sak* was present in only one isolate) and the PVL/*sea* prophage φ-IND772 (21). Among other virulence factors, the enterotoxin genes *sec* and *sel*, the gamma-hemolysin locus, *egc* cluster enterotoxins, and the enterotoxin homologue ORF CM14 were ubiquitous in ST772. We detected no statistically significant difference between core virulence factors present in the basal group and ST772-A (Fig. S3).

10.1128/mBio.01105-19.3 FIG S3 Proportion of isolates in basal strains (gray) and ST772-A (black) carrying virulence factors from the core virulence factor database, detected with ARIBA. There are no significant differences in virulence factor carriage between basal strains and ST772-A at a *P* value of <0.01 using Fisher’s exact test. Download FIG S3, PDF file, 0.2 MB. Copyright © 2019 Steinig et al. 2019 Steinig et al. This content is distributed under the terms of the Creative Commons Attribution 4.0 International license.

**FIG 4:**
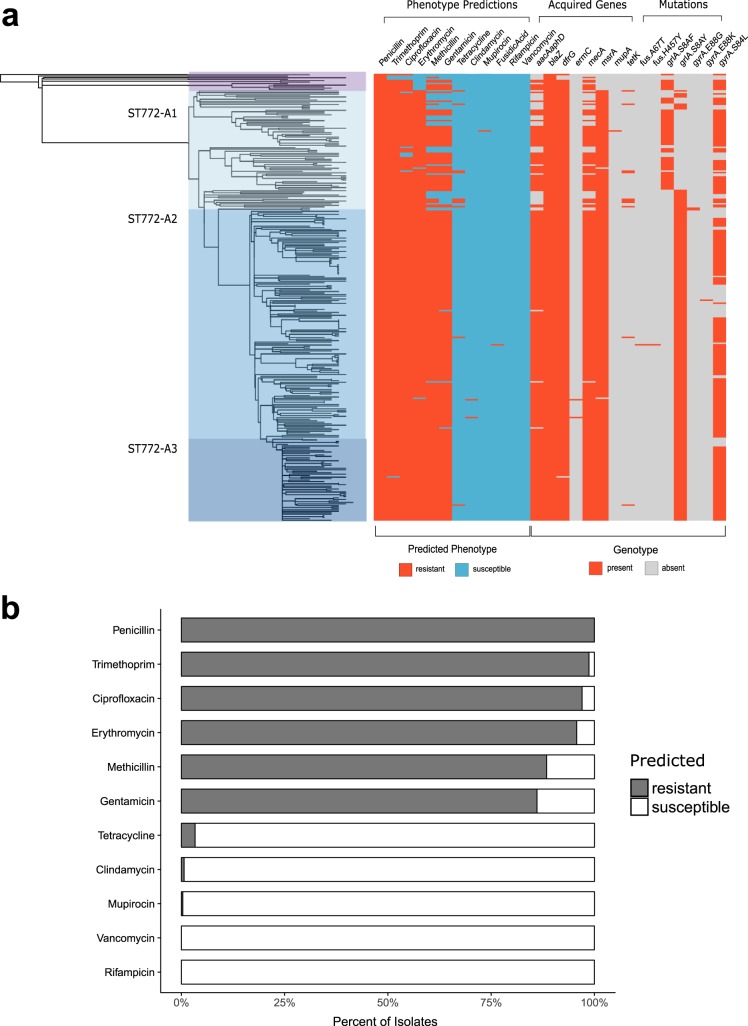
Resistome and predicted resistance phenotypes across ST772. (a) Resistome mapped to maximum likelihood phylogeny of ST772. The predicted resistant phenotype is depicted in red, while the susceptible phenotype is depicted in blue. The presence of acquired resistance genes and mutations responsible for phenotype predictions is shown in red, while the absence of these determinants is shown in gray. (b) Percentages of isolates predicted resistant (gray) or susceptible (white) for all antimicrobials included in Mykrobe Predictor.

**FIG 5:**
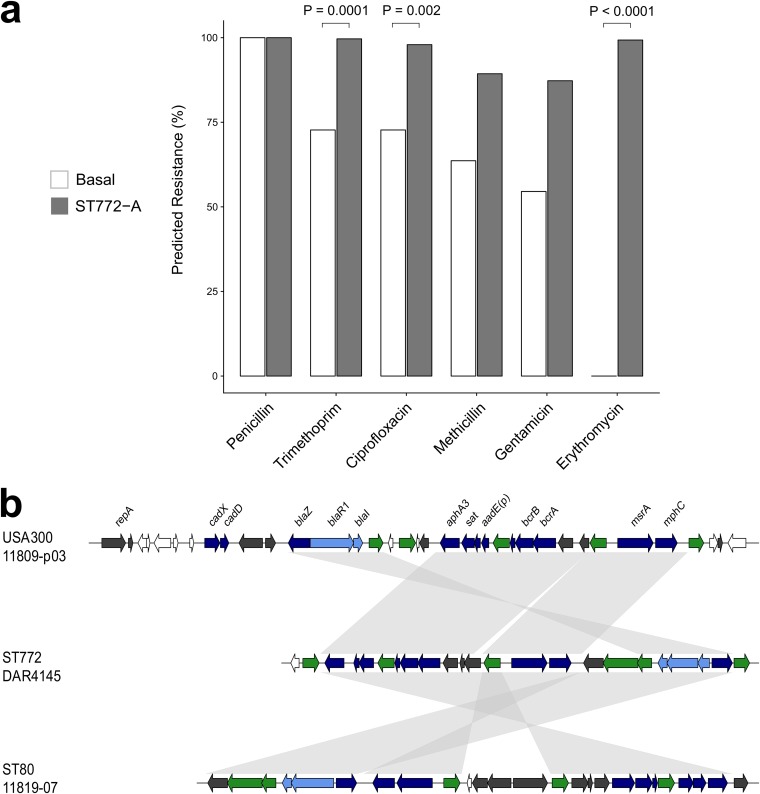
Integrated resistance plasmid in ST772. (a) Proportion of isolates predicted resistant to common antibiotics for basal isolates (*n* = 11) and isolates from ST772-A (*n* = 291). Values above bars are statistically significant differences between groups using Fisher’s exact test where *P* < 0.01. (b) BLAST comparison of the multidrug resistance plasmid in DAR4145 (middle) with the extrachromosomal plasmid 11809-p03 (top) and the SCC*mec*-IV integrated plasmid in ST80 (bottom), showing alignments of >1,000 bp and >95% nucleotide identity. The comparison highlights three regions harboring resistance genes (dark blue) and their regulators (light blue), which are flanked by transposition elements (green) and appear to have integrated with reversions and rearrangements into ST80 and ST772. Resistance genes include the β-lactam *blaZ* complex, aminoglycoside cluster *aphA3-sat4-aadE* and bacitracin resistance loci *bcrA/B*, as well as macrolide efflux genes *msrA* and *mphC*. Hypothetical proteins and genes of other annotated function are shown in white and dark gray, respectively.

We noted a pattern of increasing antimicrobial resistance as successive clades of ST772 emerged. Predicted resistance phenotypes across ST772 strains were common for ciprofloxacin (97.4%), erythromycin (96.2%), gentamicin (87.7%), methicillin (89.7%), penicillin (100%), and trimethoprim (98.8%), with a corresponding resistome composed of acquired and chromosomally carried genes and mutations (Fig. 4). There was significantly less predicted resistance in the basal strains than in ST772-A strains, including overall multidrug resistance (≥3 classes; Fisher’s exact test, 8/11 versus 291/291, *P* < 0.001) (Fig. 5a). The key resistance determinants of interest were the SCC*mec* variants, an integrated resistance plasmid, and other smaller mobile elements and point mutations.

MRSA isolates predominantly harbored one of two subtypes of SCC*mec*-V: a short variant (5C2) or a composite cassette (5C2&5), which contains a type 5 *ccr* complex including *ccrC1* (allele 8) between the *mec* gene complex and *orfX* (22) (Fig. S4). Integration of the Tn*4001* transposon carrying aminoglycoside resistance gene *aadA-aphD* occurred across isolates with different SCC*mec* types (260/267) but not in MSSA (0/35). Of the 11 isolates in the basal group, 4 were MSSA (three lacked SCC*mec* and one had a remnant SCC*mec-*IV element), and the 7 MRSA isolates all carried the larger composite cassette SCC*mec*-V (5C2&5), with 3 of these 7 strains having a variant of SCC*mec*-V (5C2&5) (Fig. 1a).

10.1128/mBio.01105-19.4 FIG S4 SCC*mec* structure of main subtypes 5C2 and 5C2&5 present in ST772, including integration of the Tn*4001* transposon in subtype 5C2. Shown is a nucleotide BLAST comparison (polygons, minimum identity >85%) of different subtypes of SCC*mec*-V. ST772 carries two subtypes, 5C2 and the composite cassette 5C2&5 downstream of *orfX* (red), which harbors an additional *ccrC* (orange). Both subtypes show the characteristic inversion of the *mec* complex (purple, orchid), flanked by complete or truncated transposition elements (green). An additional major variant of 5C2 found in the reference genome DAR4145 harbors Tn*4001* downstream of the type I restriction-modification system (RMS; yellow). The specificity subunit (*hsdS*; middle) of the RMS shows <85% nucleotide identity with other systems on SCC*mec*-V. This corresponds to unusual recognition site methylation in the ST772 PacBio reference genome DAR4145 (available at REBASE). Additional experiments will need to be conducted to match the recognition sites of all RMSs occurring in DAR4145 to the target recognition domains of *hsdS*. Download FIG S4, PDF file, 0.2 MB. Copyright © 2019 Steinig et al. 2019 Steinig et al. This content is distributed under the terms of the Creative Commons Attribution 4.0 International license.

The diversity of SCC*mec* types decreased as ST772-A diverged into subgroups (Fig. 1a and c). ST772-A1 included MSSA (*n* = 30) as well as SCC*mec*-V (5C2 [*n* = 22] and 5C2&5 [*n* = 18]) strains. Four isolates harbored a putative composite SCC element that included SCC*mec*-V (5C2), as well as *pls* and the *kdp* operon previously known from SCC*mec*-II. One isolate from the United Kingdom harbored a composite SCC*mec*-V (5C2&5) with a cadmium and zinc resistance locus (*czrC*), known from the European livestock-associated CC398-MRSA (23). Another six isolates yielded irregular and/or composite SCC elements. In contrast, the dominant subgroups ST772-A2 and -A3 exclusively carried the short SCC*mec*-V (5C2) element. Eleven of these isolates (including all isolates in NICU-2) lacked *ccrC* and two isolates carried additional recombinase genes (*ccrA/B2* and *ccrA2*). Considering the distribution of SCC*mec* across the lineage, SCC*mec* integration appears to have occurred on multiple occasions in the basal strains and ST772-A1, with subsequent modifications of SCC*mec*-V (5C2 and 5C2&2) (Fig. 1a). In contrast, integration appears to have occurred only on a single occasion at the divergence of ST772-A2 and -A3, followed by the occasional complete or partial loss of the element in these subgroups.

Predicted resistance to erythromycin was uniquely found in ST772-A and not in any of the basal strains (Fisher’s exact test, 289/291 versus 0/11, *P* < 0.001 [Fig. 5a]), characterized by the acquisition of an integrated multidrug resistance plasmid (IRP) (Fig. 5b), including the macrolide resistance locus *msrA/mphC*, as well as determinants against β-lactams (*blaZ*), aminoglycosides (*aadE-sat4-aphA3*), and bacitracin (*bcrAB*). The mosaic IRP element was highly similar to a composite extrachromosomal plasmid in ST8 (USA300) (24) and a SCC*mec* integration in the J2 region of the ST80 (25) reference genome (Fig. 5b; see also data posted at https://doi.org/10.6084/m9.figshare.8061887.v3). A search of closed S. aureus genomes (*n* = 274; October 2018) showed that the element is rare and predominantly plasmid associated across ST8 genomes (6/274), with one chromosomal integration in the ST772 reference genome and the SCC*mec* integration in the ST80 reference genome.

Three basal strains were not multidrug resistant and included two isolates from the original collections in India (RG28) and Bangladesh (NKD122) (Fig. 1a and 4a). These two strains lacked the trimethoprim determinant *dfrG* and the fluoroquinolone mutations in *grlA* or *gyrA*, including only a penicillin resistance determinant *blaZ* on a Tn*554*-like transposon. However, seven of the strains more closely related to ST772-A did harbor mobile elements and mutations conferring trimethoprim (*dfrG*) and quinolone (*grlA* and *gyrA* mutations) resistance. Interestingly, we observed a shift from the quinolone resistance *grlA* S80F mutation in basal strains and ST772-A1 to the *grlA* S80Y mutation in ST772-A2 and -A3 (Fig. 4a).

Thus, the phylogenetic distribution of the key resistance elements suggests acquisition of the IRP by a PVL-positive MSSA strain in the early 1990s (ST772-A1 divergence, 1990.02; 95% CI, 1977.5 to 1995.27), followed by fixation of both the shorter variant of SCC*mec*-V (5C2) and the *grlA* S80Y mutation in a PVL- and IRP-positive MSSA ancestor in the late 1990s (ST772-A2 divergence, 1998.25; 95% CI, 1991.81 to 2001.73) (Fig. 1a and Fig. 3).

### Canonical mutations and phenotypic comparison of basal strains and ST772-A.

We found three other mutations of interest that were present exclusively in ST772-A strains. The first mutation caused a nonsynonymous change in *fbpA* (L55P), encoding a fibrinogen-binding protein that mediates surface adhesion in S. aureus (26). The second comprised a nonsynonymous change (L67V) in the *plc* gene, encoding a phospholipase associated with survival in human blood cells and abscess environments in USA300 (27). The third encoded a nonsynonymous mutation (S273G) in *tet*(38), an efflux pump that promotes resistance to tetracyclines as well as survival in abscess environments and skin colonization (28). The functional implication of genes harboring these canonical mutations might suggest a modification of the clone’s ability to colonize and cause skin and soft tissue infections.

In light of these canonical SNPs, we selected 5 basal strains and 10 strains from ST772-A to perform a preliminary screen for potential phenotypic differences that may contribute to the success of ST772-A. We assessed *in vitro* growth, biofilm formation, cellular toxicity, and lipase activity (Fig. S6). We found no statistically significant differences between the basal strains and ST772-A in these phenotypic assays, apart from significantly lower lipase activity among ST772-A strains (Welch’s two-sided *t* test, *t* = 3.4441, degrees of freedom = 6.0004, and *P* = 0.0137), which may be related to the canonical nonsynonymous mutation in *plc*. However, it is increased rather than decreased lipase activity that has been associated with viability of S. aureus USA300 in human blood and neutrophils (27). We found no difference in the median growth rate of ST772-A compared to the basal strains (Mann-Whitney, *P* = 0.8537), although there were two ST772-A strains that grew more slowly, suggesting the possibility of some strain-to-strain variability.

## DISCUSSION

In this study, we used whole-genome sequencing in combination with epidemiological and phenotypic data to investigate the drivers behind the emergence and spread of a multidrug-resistant community-associated MRSA lineage from the Indian subcontinent. Our data suggest that the Bengal Bay clone has acquired the multidrug resistance phenotype of traditional health care-associated MRSA but retains the epidemiological characteristics of community-associated MRSA.

Emergence of a basal population of ST772 appears to have occurred on the Indian subcontinent in the early 1960s and included strains from the original isolations of ST772 in Bangladesh and India in 2004. While genomic surveillance studies from India are rare, two recent studies have detected ST772-MSSA and -MRSA in Nepal (29) and ST772-MRSA in Pakistan (30), but it is unclear whether the lineage was endemic in these countries prior to its emergence in India. Deeper genomic surveillance of ST772-MSSA and -MRSA in the region will be necessary to understand the local epidemiology and evolutionary history of the clone on the Indian subcontinent.

Establishment and expansion of a single dominant clade (ST772-A) occurred in the early 1990s and was associated with the acquisition of an integrated multidrug resistance mobile genome element (MGE). The element is similar to a previously described extrachromosomal plasmid of USA300 (24) and a partially integrated element in the SCC*mec* of an ST80 reference genome (25). While the element was found only once in the ST80 lineage (8) and occurs predominantly on plasmids in closed ST8 (USA300) genomes, its distribution and contribution to the emergence of resistance in the ST8 lineage have so far not been addressed (4, 6). In contrast, the ubiquitous occurrence and retention of the element in ST772-A suggest that it was instrumental in the emergence of the dominant clade of the Bengal Bay clone.

Furthermore, we observed a replacement of the long composite SCC*mec*-V (5C2&5) element with the shorter SCC*mec-*V (5C2) and fixation of the quinolone resistance mutation from *grlA* S80F to the *grlA* S80Y as ST772-A2 and -A3 became the dominant population subgroups in the 1990s. In light of earlier studies demonstrating a fitness advantage in having a smaller SCC*mec* element (31–33), the fixation of the shorter SCC*mec*-V (5C2) may be a contributing factor to the success of ST772.

We observed a lack of significant differences in growth between basal strains and ST772-A. This may suggest that acquisition of drug resistance was not accompanied by a major fitness cost to ST772-A and raises the possibility that members of this clade will survive in environments where antibiotics are heavily used, such as hospitals or in communities with poor antibiotic stewardship, but may also be at little disadvantage in environments where there is less antibiotic use. Notably, competitive-fitness experiments involving the community-associated MRSA lineages USA300 and ST80 revealed that the biological cost of resistance to methicillin, fusidic acid, and fluoroquinolones is reversed in the presence of trace amount of antibiotics (34). In regions such as the Indian subcontinent, where community use of antibiotics is not well regulated, it is plausible that lineages such as ST772-A could thrive in such environments. While our results are consistent with those of Gustave et al. (34), it should be noted that our phenotypic assays assessed only a small number of isolates and the growth assays were conducted *in vitro* and under nutrient-rich conditions that are unlikely to capture anything but very high fitness costs. Future studies may build on this preliminary work and consider a more in-depth analysis of a larger number of isolates.

Given the available epidemiological data, phylogeographic heterogeneity, and the clone’s limited success to establish itself in regions outside its range of endemicity in South Asia (Fig. 1a), there appears to be ongoing exportation of ST772 from the Indian subcontinent, associated with travel and family background in the region. This is supported by reports of MRSA importation in travelers, including direct observations of ST772 importation by returnees from India (35). Our data suggest nonendemic spread within households and the community, including short-term outbreaks at two NICUs in Ireland. This pattern of limited endemic transmission is consistent with reports of small transmission clusters in hospitals and households during a comprehensive surveillance study of ST772 in Norway (16).

There have been small numbers of ST772 isolates identified from other countries and regions of the world not included in this study, including Austria, Finland and Slovenia, Taiwan, Japan, China, and Nigeria (36–43). These studies have generally been broader descriptions of circulating MRSA genotypes, of which only a small minority have been found to be ST772. These reports would therefore support similar patterns of either sporadic importation or short-lived chains of local transmission of ST772 as observed for isolates in our study.

Overall, the pattern of spread mirrors those of other community-associated MRSA lineages, such as USA300 (5, 44), ST80-MRSA (8), and ST59 (7), where clones emerge within a particular geographic region, are exported elsewhere, but rarely become established and endemic outside their place of origin. In contrast, health care-associated MRSA clones such as CC22-MRSA-IV (EMRSA-15) (45) and ST239-MRSA-III (46, 47) demonstrate much stronger patterns of phylogeographic structure, consistent with importation into a country followed by local dissemination through the health care system. While there are indications for resistance acquisition driving regional community-asscoiated lineages, such as the Taiwan clade of ST59 (7), we found strong indications in our study that the acquisition of mutations and mobile elements associated with multidrug resistance was the dominant driver behind the emergence of the Bengal Bay clone on the Indian subcontinent and its subsequent intercontinental transmission. Moreover, we observed an unusual, near complete lack of phylogeographic structure in the population, compared to other previously investigated community-associated clones, providing evidence for ongoing circulation and exportation from the Indian subcontinent followed by limited endemic transmission.

Our data trace the evolution of the Bengal Bay clone on the Indian subcontinent, where it emerged in the 1960s and diverged into a single dominant clade in the 1990s. Its rapid emergence may have been driven by the dissemination of mobile genetic elements, particularly those that confer drug resistance, such as the acquisition of a multidrug resistance integrated plasmid and variants of SCC*mec*. Patient epidemiology and phylogenetic heterogeneity suggest a pattern of ongoing exportation from the Indian subcontinent and limited endemic transmission after importation. The Bengal Bay clone therefore appears to combine the epidemiological characteristics of community-associated MRSA lineages with an unusually resistant genotype traditionally seen in health care-associated MRSA.

Considering the widespread use of antibiotics and associated poor antibiotic regulation, limited public health infrastructure, and high population density in parts of South Asia, the emergence and global dissemination of multidrug-resistant bacterial clones (both Gram positive and Gram negative) are alarming and perhaps not surprising. Global initiatives and funding to monitor the occurrence of emerging clones and resistance mechanisms, and support for initiatives in antimicrobial stewardship at community, health care, and agricultural levels, are urgently needed.

## MATERIALS AND METHODS

### Isolates.

Isolates were obtained from Australia (21 isolates), Bangladesh (3), Denmark (70), England (103), Germany (16), Hong Kong (6), India (44), Ireland (28), Italy (2), the Netherlands (4), New Zealand (17), Norway (3), Saudi Arabia (1), Scotland (29), and the United Arab Emirates (1) between 2004 and 2012 (see data posted at https://doi.org/10.6084/m9.figshare.8061887.v3). The collection was supplemented with six previously published genome sequences from India (21, 22, 48). Notable samples include the initial isolates from Bangladesh and India (10, 11), two hospital-associated (NICU) clusters from Ireland (20), and isolates from a single health care worker at a veterinary clinic sampled over two consecutive weeks (VET) (49). Geographic regions were designated Australasia (Australia and New Zealand), East Asia (Hong Kong), South Asia (India, Bangladesh), Arabian Peninsula (Saudi Arabia and United Arab Emirates), and Europe (Denmark, England, Germany, Ireland, Italy, the Netherlands, Norway, and Scotland).

### Clinical data and epidemiology.

Anonymized patient data were obtained for the date of collection, clinical symptoms, geographic location, epidemiological connections based on family or travel history, and acquisition in nosocomial or community environments, where available. Clinical symptoms were summarized as skin and soft tissue infections (abscesses, boils, ulcers, exudates, pus, and ear and eye infections), urogenital (vaginal swabs and urine), bloodstream (bacteremia), or respiratory infections (pneumonia and lung abscesses), and colonization (swabs from ear, nose, throat, perineum, or the environment) (Fig. S5). Literature and sample maps (see data posted at https://doi.org/10.6084/m9.figshare.8061887.v3) were constructed with *geonet*, a wrapper for geographic projections with Leaflet in R (https://github.com/esteinig/geonet).

10.1128/mBio.01105-19.5 FIG S5 Isolation and acquisition in ST772. (a) Source of isolation as described in Materials and Methods; data are available at https://doi.org/10.6084/m9.figshare.8061887.v3. Skin and soft tissue infections (SSTIs) and colonization are the most prevalent presentations of ST772 (-, no data). (b) Acquisition of ST772 isolates (HA, health care associated; CA, community associated; CO, community onset; HO, health care onset; -, no data). Data of acquisition status are sparse, and further studies may endeavor to collect reliable epidemiological data to map the circulation of the Bengal Bay clone in health care and community environments. Download FIG S5, PDF file, 0.03 MB. Copyright © 2019 Steinig et al. 2019 Steinig et al. This content is distributed under the terms of the Creative Commons Attribution 4.0 International license.

10.1128/mBio.01105-19.6 FIG S6 Phenotypic assays for representative strains from the basal group (white, *n* = 5) and ST772-A (gray, *n* = 10) for optical density measurements (595 nm) of biofilm formation, accounting for day-to-day variability relative to control strain E-MRSA15 (percent) (a), overnight growth in tryptic soy broth (doubling time per minute) measured by optical density (600 nm) (b), cytotoxicity of neat (100%) and diluted (30%) bacterial supernatant to THP-1 cells measured as cell death by flow cytometry (c), absorbance measurements (404 nm) of erythrocyte hemolysis in neat (100%) and diluted (30%) bacterial supernatant (d), and lipase activity of *para*-nitrophenyl butyrate (pNPB) or *para*-nitrophenyl palmitate (pNPP) (release of pNP per minute) in neat bacterial supernatant measured by absorbance (410 nm) (e). Slow-growing strains H104580604 and HPAS101177P were considered outliers and removed from the growth box plot for visual clarity after calculation of median and interquartile ranges and assessment of significance. Error bars show standard errors; the asterisk indicates a significant difference in pNPP release (Welch’s two-sided *t* test, *t* = 3.4441, df = 6.0004, and *P* = 0.0137) between basal strains and ST772. Download FIG S6, PDF file, 0.08 MB. Copyright © 2019 Steinig et al. 2019 Steinig et al. This content is distributed under the terms of the Creative Commons Attribution 4.0 International license.

Where available, acquisition in community or health care environments was recorded in accordance with guidelines from the CDC. Community-associated MRSA is therein classified as an infection in a person who has none of the following established risk factors for MRSA infection: isolation of MRSA more than 48 h after hospital admission; history of hospitalization, surgery, dialysis, or residence in a long-term-care facility within 1 year of the MRSA culture date; the presence of an indwelling catheter or a percutaneous device at the time of culture; or previous isolation of MRSA (50, 51) (Fig. S5).

A valid epidemiological link to South Asia was declared if either travel or family background could be reliably traced to Bangladesh, India, Nepal, or Pakistan. If both categories (travel and family) were unknown or where data were available for only one category and did not show a link to the region, we conservatively declared the link as unknown or absent, respectively. The longitudinal collection (*n* = 39) from a staff member at a veterinary hospital in England was treated as a single patient sample.

### Sequencing, quality control, and assembly.

Unique index-tagged libraries were created for each isolate, and multiplexed libraries were sequenced on an Illumina HiSeq with 100-bp paired-end reads. Samples from the veterinary staff member were processed and sequenced as described by Paterson et al. (49). Read quality control was conducted with Trimmomatic (52), Kraken (53), and FastQC (https://www.bioinformatics.babraham.ac.uk/projects/fastqc). Quality control identified a large proportion of reads classified as Enterococcus faecalis in sample HWM2178. *In silico* microarray typing (see below) identified an additional 13 isolates with possible intraspecific contamination due to simultaneous presence of *agr I* and *II*, as well as capsule types 5 and 8. We excluded these isolates from all genomic analyses. Raw Illumina data were subsampled to 100× coverage and assembled with the SPAdes (54) pipeline Shovill (https://github.com/tseemann/shovill), which wraps SPAdes, Lighter (55), FLASH (56), BWA MEM (57), SAMtools (58), KMC (59), and Pilon (60). Final assemblies were annotated with Prokka v1.11 (61).

### MLST and SCC typing.

*In silico* multilocus sequence typing (MLST) was conducted using mlst (https://github.com/tseemann/mlst) on the assembled genomes with the S. aureus database from PubMLST (https://pubmlst.org/saureus/). Three single-locus variants (SLVs) of ST772 were detected and retained for the analysis, describing ST1573, ST3362, and ST3857. Sequences of experimentally verified sets of probes for SCC-related and other S. aureus-specific markers (62, 63) were BLAST searched against SPAdes assemblies (*in silico* microarray typing), allowing prediction of the presence or absence of these markers and detailed typing of SCC elements. We assigned MRSA to four isolates that failed precise SCC classification based on the presence of *mecA* on the probe array and detection of the gene with Mykrobe Predictor (64).

### Variant calling.

Samples passing quality control (*n* = 340) were aligned to the PacBio reference genome DAR4145 from Mumbai, and variants were called with the pipeline Snippy (available at https://github.com/tseemann/snippy), which wraps BWA MEM, SAMtools, SnpEff (65), and Freebayes (66). Core SNPs were defined as being present in all samples (ignoring insertions and deletions, *n* = 7,063) and were extracted with *snippy-core* at default settings. We assigned canonical SNPs for ST772-A as those present exclusively in all isolates of ST772-A but not in the basal strains. Annotations of variants were based on the reference genome DAR4145.

### Phylogenetics and recombination.

A maximum likelihood (ML) tree under the general time reversible (GTR) model of nucleotide substitution with among-site rate heterogeneity across 4 categories (GTR + Γ), ascertainment bias correction (Lewis), and 100 bootstrap (BS) replicates was generated based on 7,063 variant sites (core genome SNPs) in RaxML-NG 0.5.0 (available at https://github.com/amkozlov/raxml-ng), which implements the core functionality of RAxML (67). The tree with the highest likelihood out of 10 replicates was midpoint rooted and visualized with interactive Tree of Life (ITOL) (Fig. 1a and 2a and Fig. S2 and S7B) (68). In all phylogenies (Fig. 1a, Fig. 2a, and Fig. 3 and Fig. S2, S7, and S8), samples from the veterinary staff member were collapsed for clarity.

10.1128/mBio.01105-19.7 FIG S7 (A) Population network graph of ST772. (a) Plot of the number of detected communities (n) versus the number of mutual nearest neighbors (*k*) in NetView (*k* = 1 to 100) using a pairwise Hamming distances and community detection algorithms as implemented in *igraph* and *NetView* v.1.1 (http://github.com/esteinig/netview). While community detection differs strongly in the assembly phase of the network (*k* = 1 to 25), the plot indicates a relative congruence of community detection between algorithms at *k* values of >25 to 40. (b) Complete network topology (Fruchtermann-Reingold) at a *k* value of 40, showing communities from fast-greedy modularity optimisation (polygons) and SCC*mec* type as in Fig. 1a. The veterinary cluster, excluded from the main network in Fig. 1c, is shown in this representation (gray polygon, VET). (B) Maximum likelihood phylogenies of ST772 showing community clusters detected with NetView (colors) and their bootstrap support values in the midpoint rooted main phylogeny, with black circles indicating bootstrap support >95% (a), and as unrooted phylogeny, demonstrating a pattern of rapid population divergence and proliferation of ST772-A (b). Download FIG S7, PDF file, 0.5 MB. Copyright © 2019 Steinig et al. 2019 Steinig et al. This content is distributed under the terms of the Creative Commons Attribution 4.0 International license.

10.1128/mBio.01105-19.8 FIG S8 Pan-genome of ST772 computed from assembled and Prokka-annotated genomes (*n* = 339, excluding reference genome DAR4145) using Roary. The lineage demonstrates a stable core genome (genes occurring in >98% of strains) with little variation in the accessory genome. Pie chart and contiguous visualization were determined across the core genome phylogeny, using *roary_plots.py* (https://github.com/sanger-pathogens/Roary/contrib/roary_plots). Accessory genes comprise mainly phage-associated coding regions, plasmid-associated genes, and specific genes rarely observed across the population of ST772, such as a type I RMS, lipoprotein-like genes, cassette recombinases, and fibronectin binding protein A (data available at the repository). Download FIG S8, PDF file, 0.09 MB. Copyright © 2019 Steinig et al. 2019 Steinig et al. This content is distributed under the terms of the Creative Commons Attribution 4.0 International license.

A confirmation alignment (*n* = 351) was computed as described above for resolving the pattern of divergence in the basal strains of ST772. The alignment included the CC1 strain MW2 as an outgroup, as well as another known SLV of CC1, sequence type 573 (*n* = 10). The resulting subset of core SNPs (*n* = 25,701) was used to construct an ML phylogeny with RaxML-NG (GTR + Γ) and 100 bootstrap replicates (Fig. S1A). We also confirmed the general topology of our main phylogeny as described above using the core genome alignment of 2,545,215 nucleotides generated by Snippy, masking sites if they contained missing (−) or uncertain (N) characters across ST772. Phylogenies were visualized using ITOL, *ape* (69), *phytools* (70), *ggtree* (71), or *plotTree* (https://github.com/katholt/plotTree). Patristic distances to the root of the phylogeny (Fig. S1C) were computed in the *adephylo* (72) function *distRoot*.

### Dating analysis.

We removed sites with missing data (−) from the core alignment generated by Snippy and then ran Gubbins (73) to detect homologous recombination events, using a maximum of five iterations and the GTR + Γ model in RaxML. Following the removal of SNPs related to recombination there were 6,907 SNPs in the alignment. We used Least Squares Dating (LSD) v0.3 (74) to obtain a time-scaled phylogenetic tree. This method fits a strict molecular clock to the data using a least-squares approach. Importantly, LSD does not explicitly model rate variation among lineages, and it does not directly account for phylogenetic uncertainty. However, its accuracy is similar to that obtained using more sophisticated Bayesian approaches (75), with the advantage of being computationally less demanding.

LSD typically requires a phylogenetic tree with branch lengths in substitutions per site and calibrating information for internal nodes or for the tips of the tree. We used the phylogenetic tree inferred using maximum likelihood in PhyML (76) (after removing recombination with Gubbins, as described above) using the GTR + Γ substitution model with 4 categories for the Γ distribution. We used a combination of nearest-neighbor interchange and subtree-prune-regraft to search tree space. Because PhyML uses a stochastic algorithm, we repeated the analyses 10 times and selected that with the highest phylogenetic likelihood. To calibrate the molecular clock in LSD, we used the collection dates of the samples (i.e., heterochronous data). The position of the root can be specified *a priori*, using an outgroup or by optimizing over all branches. We chose the latter approach. To obtain uncertainty around node ages and evolutionary rates, we used the parametric bootstrap approach with 100 replicates implemented in LSD.

An important aspect of analyzing heterochronous data is that the reliability of estimates of evolutionary rates and timescales is contingent on whether the data have temporal structure. In particular, a sufficient amount of genetic change should have accumulated over the sampling time. We investigated the temporal structure of the data by conducting a regression of the root-to-tip distances of the maximum likelihood tree as a function of sampling time (77) and performing a date randomization test (78). Under the regression method, the slope of the line is a crude estimate of the evolutionary rate, and the extent to which the points deviate from the regression line determines the degree of clocklike behavior, typically measured using R (79). The date randomization test consists of randomizing the sampling times of the sequences and reestimating the rate each time. The randomizations correspond to the distribution of rate estimates under no temporal structure. Consequently, the data have strong temporal structure if the rate estimate using the correct sampling times is not within the range of those obtained from the randomizations (80). We conducted 100 randomizations, which suggested strong temporal structure for our data (Fig. S1D). We also verified that the data did not display phylogenetic-temporal clustering, a pattern which sometimes misleads the date randomization test (81).

### Nucleotide diversity.

Pairwise nucleotide diversity and SNP distance distributions for each region with >10 samples (Australasia, Europe, South Asia) were calculated as outlined by Stucki et al. (82). Pairwise SNP distances were computed using the SNP alignment from Snippy (*n* = 7,063) and the *dist.dna* function from *ape*, with raw counts and deletion of missing sites in a pairwise fashion. An estimate of average pairwise nucleotide diversity per site (π) within each geographic region was calculated from the SNP alignments using raw counts divided by the alignment length. Confidence intervals for each region were estimated using 1,000 bootstrap replicates across nucleotide sites in the original alignment via the *sample* function (with replacement) and the 2.5% to 97.5% quantile range (Fig. 2b).

### Population structure.

We used the network analysis and visualization tool NetView (83, 84) (available at http://github.com/esteinig/netview) to delineate population subgroups in ST772. Pairwise Hamming distances were computed from the core SNP alignment derived from Snippy. The distance matrix was used to construct mutual *k*-nearest-neighbor networks from *k *= 1 to *k *= 100. We ran three commonly used community detection algorithms as implemented in *igraph* to limit the parameter choice to an appropriate range for detecting fine-scale population structure: fast-greedy modularity optimization (85), Infomap (86), and Walktrap (87). We thereby accounted for differences in the mode of operation and resolution of algorithms. Plotting the number of detected communities against *k*, we were able to select a parameter value at which the results from the community detection were approximately congruent (Fig. S7A).

Since we were interested in the large-scale population structure of ST772, we selected a *k *value of 40 and used the low-resolution fast-greedy modularity optimization to delineate final population subgroups. Community assignments were mapped back to the ML phylogeny of ST772 (Fig. 1a). All subgroups agreed with the phylogenetic tree structure and were supported by ≥99% bootstrap values (Fig. S7B). One exception was isolate HW_M2760, located within ST772-A2 by phylogenetic analysis but assigned to ST772-A3 by network analysis (Fig. S7A and B). This appeared to be an artifact of the algorithm, as its location and connectivity in the network representation matched its phylogenetic position within ST772-A2. The network and communities were visualized using the Fruchtermann-Reingold algorithm (Fig. 1c), excluding samples from the veterinary staff member in Fig. 1c (Fig. S7A).

### Local transmission clusters.

We obtained approximate transmission clusters by employing a network approach supplemented with the ML topology and patient data, including date of collection, location of collection, and patient family links and travel or family links to South Asia. We used pairwise SNP distances to define a threshold of 4 SNPs, corresponding to the maximum possible SNP distance obtained within 1 year under a core genome substitution rate of 1.61 × 10^−6^ nucleotide substitutions/site/year. We then constructed the adjacency matrix for a graph, in which isolates were connected by an undirected edge, if they had a distance of less than or equal to 4 SNPs. All other isolates were removed from the graph, and we mapped the resulting connected components to the ML phylogeny, showing that in each case the clusters were also reconstructed in the phylogeny, where isolates diverged from a recent common ancestor (gray highlights in Fig. 2a and Fig. S2). We then traced the identity of the connected components in the patient metadata and added this information to each cluster. NICU clusters were reconstructed under these conditions (Fig. 2a and Fig. S2).

### Antimicrobial resistance, virulence factors, and pan-genome analysis.

Mykrobe Predictor was employed for antibiotic susceptibility prediction and detection of associated resistance determinants and mutations. Mykrobe Predictor has a demonstrated sensitivity and specificity >99% for calling phenotypic resistance and is comparable to gold-standard phenotyping in S. aureus (64). Predicted phenotypes were therefore taken as a strong indication for actual resistance phenotypes in ST772. Genotype predictions also reflect multidrug resistance profiles (aminoglycosides, β-lactams, fluoroquinolones, macrolides-lincosamides-streptogramin B [MLS], and trimethoprim) reported for this clone in the literature (16–20, 88). As most resistance-associated MGEs in the complete reference genome DAR4145 are mosaic-like and flanked by repetitive elements (18), we used specific diagnostic genes present as complete single copies in the reference annotation of DAR4145 (18) to define the presence of the IRP (*msrA*) and Tn*4001* (*aacA-aphD*). Mykrobe Predictor simultaneously called the *grlA* mutations S80F and S80Y for quinolone resistance phenotypes. However, in all cases one of the variants was covered at an extremely low median k-mer depth (<20), and we consequently assigned the variant with a higher median k-mer depth at *grlA* (see data posted at https://doi.org/10.6084/m9.figshare.8061887.v3).

ARIBA (89) with default settings and the core Virulence Factor database were used to detect the complement of virulence factors in ST772. We corroborated and extended our results with detailed *in silico* microarray typing, including the presence of the *egc* gene cluster or S. aureus-specific virulence factors such as the enterotoxin homologue ORF CM14. Differences in detection of relevant virulence factors between the *in silico* typing and ARIBA included, among others, *lukS/F-PVL* (337 versus 336), *sea* carried on the φ-IND772 prophage (336 versus 326), *sec* (333 versus 328), and *sak* (1 versus 2). Since *in silico* microarray typing was based on assembled genomes and may therefore be prone to assembly errors, we used results from the read-based typing with ARIBA to assess statistical significance of virulence factors present in basal strains and ST772-A (Fig. S3).

Pan-genome analysis was conducted using Prokka-annotated assemblies in Roary (90), with minimum protein BLAST identity at 95% and minimum percentage for a gene to be considered core at 99% (Fig. S8). A gene synteny comparison between major SCC*mec* types was plotted with genoPlotR (91) (Fig. S4). A nucleotide BLAST comparison between the extrachromosomal plasmid 11809-03 of USA300, the integrated resistance plasmid in the ST772 reference genome DAR4145, and the integrated plasmid region in strain 11819-07 of ST80 was plotted with geneD3 (https://github.com/esteinig/geneD3/), showing segments of >1 kb (see data posted at https://doi.org/10.6084/m9.figshare.8061887.v3).

We searched for the three resistance regions which aligned to the 11819-07 and the 11809-03 plasmid (DAR4145 reference genome; R1, 1,456,024 to 1,459,959 bp; R2, 1,443,096 to 1,448,589 bp; and R3, 1,449,679 to 1,453,291 bp) in all completed S. aureus genomes (including plasmids) in RefSeq (NCBI) and the NCTC3000 project (http://www.sanger.ac.uk/resources/downloads/bacteria/nctc/) using nctc-tools (https://github.com/esteinig/nctc-tools) and nucleotide BLAST with a minimum of 90% coverage and identity (*n* = 273). Since the IRP is mosaic-like and composed of several mobile regions, we retained query results only if all three of the regions were detected. We then traced the integration sites in the accessions, determining whether integrations occurred in the chromosome or plasmids. Multilocus sequence types were assigned using *mlst* (https://github.com/tseemann/mlst).

### Growth curves.

S. aureus strains were grown overnight in 5 ml of tryptic soy broth (TSB; Fluka) with shaking (180 rpm) at 37°C. Overnight cultures were diluted 1:1,000 in fresh TSB, and 200 μl was added to a 96-well plate (Costar) in triplicate. Growth was measured 37°C with shaking (300 rpm) using a FLUOROstar fluorimeter (BMG Labtech) at an absorbance wavelength of 600 nm. Growth curves represent the means of triplicate results.

### Cell culture conditions.

The monocyte/macrophage THP-1 cell line was maintained in suspension in 30 ml of RPMI 1640 medium supplemented with 10% heat-inactivated fetal bovine serum (FBS), 1 μM l-glutamine, 200 U/ml of penicillin, and 0.1 mg/ml of streptomycin at 37°C in a humidified incubator with 5% CO_2_. Cells were harvested by centrifugation at 700 × *g* for 10 min at room temperature and resuspended to a final density of 1 × 10^6^ to 1.2 × 10^6^ cells/ml in tissue-grade phosphate-buffered saline (PBS), typically yielding >95% viable cells as determined by easyCyte flow cytometry (Millipore).

Human erythrocytes were harvested from 10 ml of human blood following treatment in sodium heparin tubes (BD). Whole blood was centrifuged at 500 × *g* for 10 min at 4°C. Supernatant (plasma) was aspirated and cells were washed twice in 0.9% NaCl and centrifuged at 700 × *g* for 10 min. The cell pellet was gently resuspended in 0.9% NaCl and diluted to 1% (vol/vol).

### Cytotoxicity assay.

To monitor S. aureus toxicity, S. aureus strains were grown overnight in TSB, diluted 1:1,000 in 5 ml of fresh TSB, and grown for 18 h at 37°C with shaking (180 rpm). Bacterial supernatants were prepared by centrifugation of 1 ml of bacterial culture at 20,000 × *g* for 10 min. For assessing toxicity to THP-1 cells, 20 μl of cells was incubated with 20 μl of bacterial supernatant and incubated for 12 min at 37°C. Both neat and 30% diluted supernatant (in TSB) were used, as certain S. aureus strains were considerably more toxic than others. Cell death was quantified using easyCyte flow cytometry using the Guava viability stain according to the manufacturer’s instructions. Experiments were done in triplicate. For assessing hemolysis, 150 μl of 1% (vol/vol) erythrocytes were incubated with 50 μl of either neat or 30% supernatant in a 96-well plate for 30 min at 37°C. Plates were centrifuged for 5 min at 300 × *g*, 75 μl of supernatant was transferred to a new plate, and absorbance was measured at 404 nm using a FLUOROstar fluorimeter (BMG Labtech). Normalized fluorescence was achieved using the equation (*A_t_* – *A*_0_)/(*A_m_*/*A*_0_), where *A_t_* is the hemolysis absorbance value of a strain, *A*_0_ is the minimum absorbance value (negative control of 0.9% NaCl), and *A_m_* is the maximum absorbance value (positive control of 1% Triton X-100).

### Lipase assay.

Bacterial supernatants used in the above-described cytotoxicity assays were also used to assess lipase activity, using the protocol published by Cadieux et al. (92), with modifications. Briefly, 8 mM *para*-nitrophenyl butyrate (pNPB), the short-chain substrate, or *para*-nitrophenyl palmitate (pNPP), the long-chain substrate (Sigma), was mixed with a buffer (50 mM Tris-HCl [pH 8.0], 1 mg/ml of gum arabic, and 0.005% Triton X-100) in a 1:9 ratio to create assay mixtures. A standard curve using these assay mixtures and *para*-nitrophenyl (pNP) (Sigma) was created, and 200 μl of each dilution was pipetted into 1 well of a 96-well plate (Costar). A total of 180 μl of each assay mixture was pipetted into the remaining wells of a 96-well plate, and 20 μl of the harvested bacterial supernatant was mixed into the wells. The plate was placed in a FLUOstar Omega microplate reader (BMG Labtech) at 37°C, and a reading at 410 nm was taken every 5 min for 1 h. The absorbance readings were converted to micromolar concentration of pNP released per minute using the standard curve.

### Biofilm formation.

Semiquantitative measurements of biofilm formation on 96-well, round-bottom polystyrene plates (Costar) were made based on the classical, crystal violet method of Ziebuhr et al. (93). Eighteen-hour bacterial cultures grown in TSB were diluted 1:40 into 100 μl of TSB containing 0.5% glucose. Perimeter wells of the 96-well plate were filled with sterile H_2_O, and plates were placed in a separate plastic container inside a 37°C incubator and grown for 24 h under static conditions. Following 24 h of growth, plates were washed five times in PBS, dried, and stained with 150 μl of 1% crystal violet for 30 min at room temperature. Following five washes of PBS, wells were resuspended in 200 μl of 7% acetic acid, and optical density at 595 nm was measured using a FLUOROstar fluorimeter (BMG Labtech). To control for day-to-day variability, a control strain (E-MRSA15) was included on each plate in triplicate, and absorbance values were normalized against this. Experiments were done using six technical repeats from 2 different experiments.

### Statistical analysis.

All statistical analyses were carried out in R or python and considered significant at a *P* value of <0.05, except for comparisons of proportions across the multiple virulence and resistance elements, which we considered be statistically significant at a *P* value of <0.01. Veterinary samples (*n* = 39) were restricted to one isolate (one patient, Staff_E1A) for statistical comparison of region of isolation, proportion of resistance, virulence, and MSSA between basal strains and ST772-A (*n* = 302; see above [Fig. 5a and Fig. S3]). Differences in pairwise SNP distance and nucleotide diversity between all regions were assessed using nonparametric Kruskal-Wallis test and *post hoc* Dunn’s test for multiple comparisons with Bonferroni correction, as distributions were assumed to be not normal (*n* = 340 [Fig. 2b and Fig. S1B]). Phenotypic differences were assessed for normality with Shapiro-Wilk tests. We consequently used either Welch’s two-sided *t* test or the nonparametric two-sided Wilcoxon rank sum test (Fig. S6).

### Data availability.

Supplemental data tables and interactive files include a summary of publication and isolates used in this study, raw data from various analyses, and measurement of phenotype experiments, as well as interactive maps and gene comparisons. Supplemental data tables and files, with the exception of supplemental figures, are hosted on Figshare (https://doi.org/10.6084/m9.figshare.8061887.v3). Core analyses, including parameter settings, cluster resource configurations, and versioned software distributions, are reproducible through the *bengal-bay-0.1* workflow, which can be found along with other scripts and data files at our GitHub repository (https://github.com/esteinig/ST772). The workflow is implemented in Snakemake (94) and runs in virtual environments that include software distributed in the Bioconda (95) channel. Analyses were conducted on the Cheetah cluster at the Menzies School of Health Research, Darwin, Australia.

Short-read sequences have been deposited at ENA under project accession PRJEB3201. Additional isolates from India are available from the SRA under accession numbers SRR404118, SRR653209, SRR653212, and SRR747869 to SRR747873. Outgroup strains used in the context phylogeny are available from ENA under accession numbers SRR592258 (MW2), ERR217298, ERR217349, ERR221806, ERR266712, ERR279022, ERR279023, ERR278908, ERR279026, ERR716976, and ERR717011 (ST573). The ST772 reference genome DAR4145 is available at GenBank under accession number CP010526.1.
