# Supplementary figures and images for: Evolution and Global Transmission of a Multidrug-Resistant, Community-Associated Methicillin-Resistant Staphylococcus aureus Lineage from the Indian Subcontinent
Source: mBio. 2019 Nov 26;10(6):e01105-19. doi: 10.1128/mBio.01105-19 (PMC6879714; doi:10.1128/mBio.01105-19)

A

a

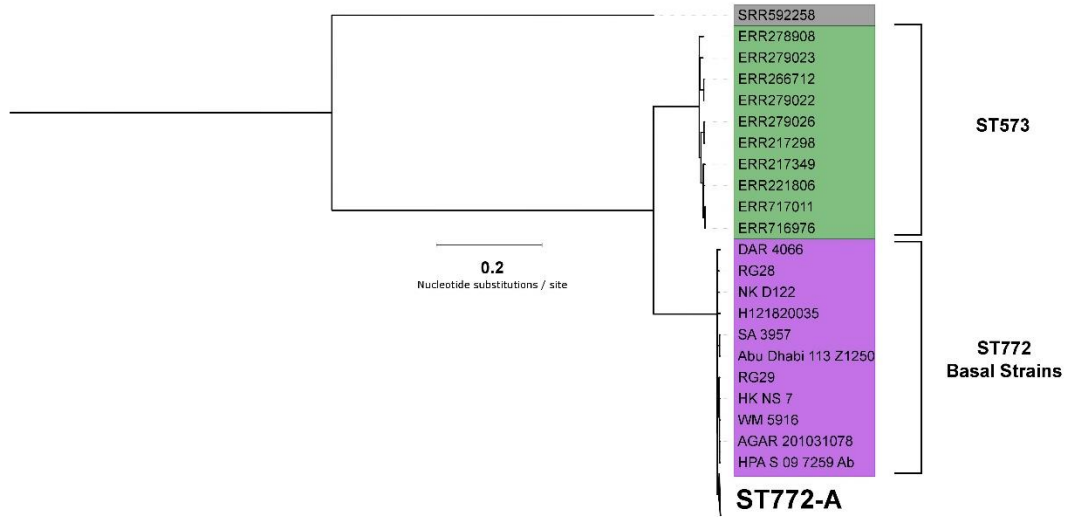

b

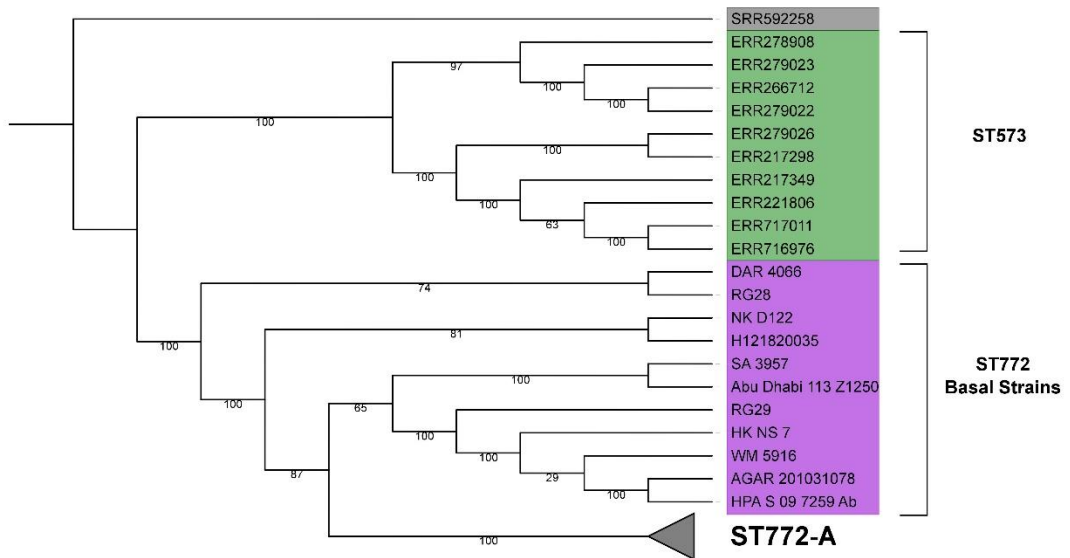

**B**

**a**

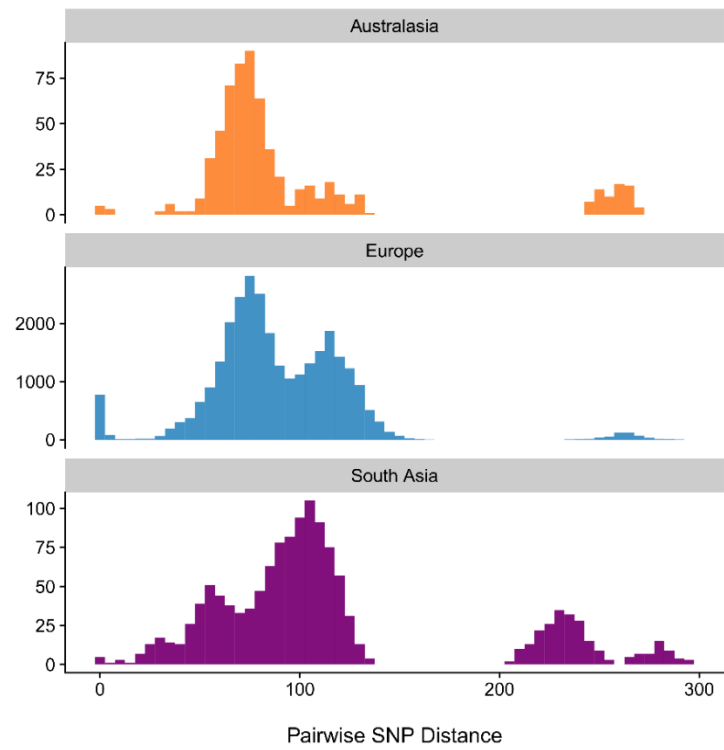

**b**

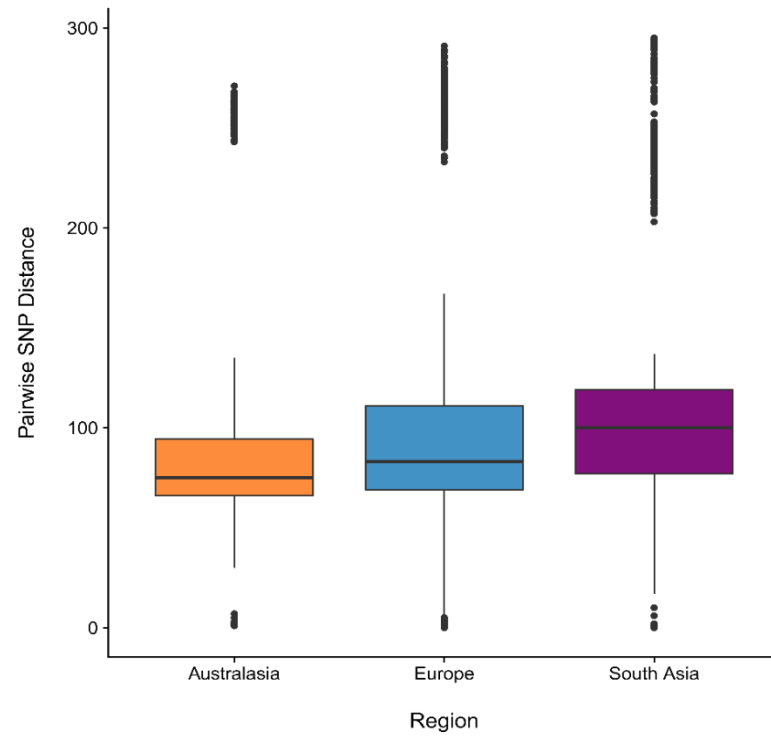

**C**

**a**

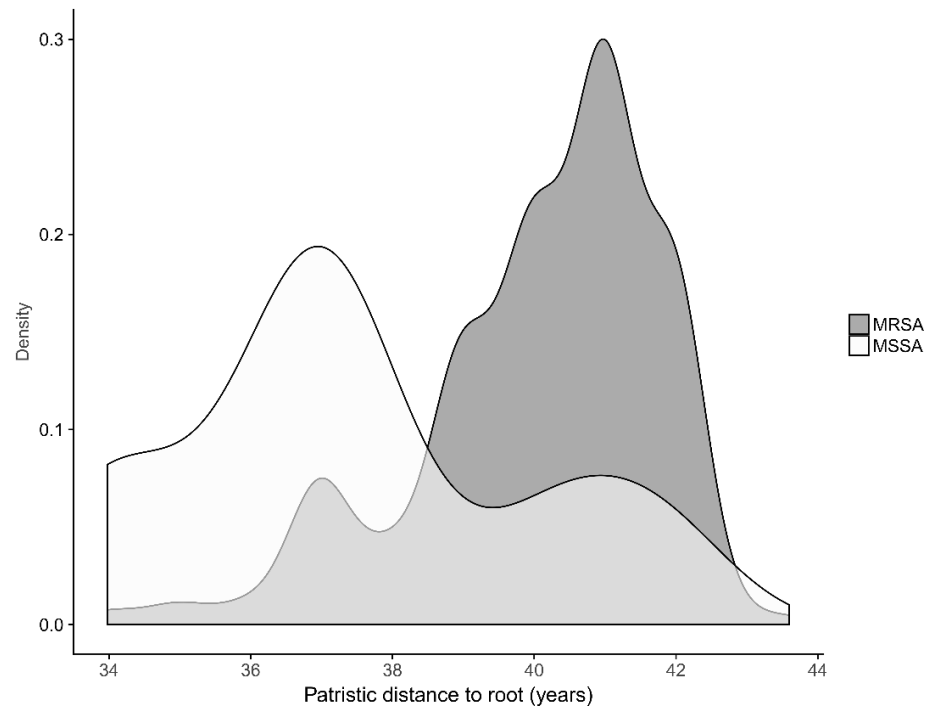

**b**

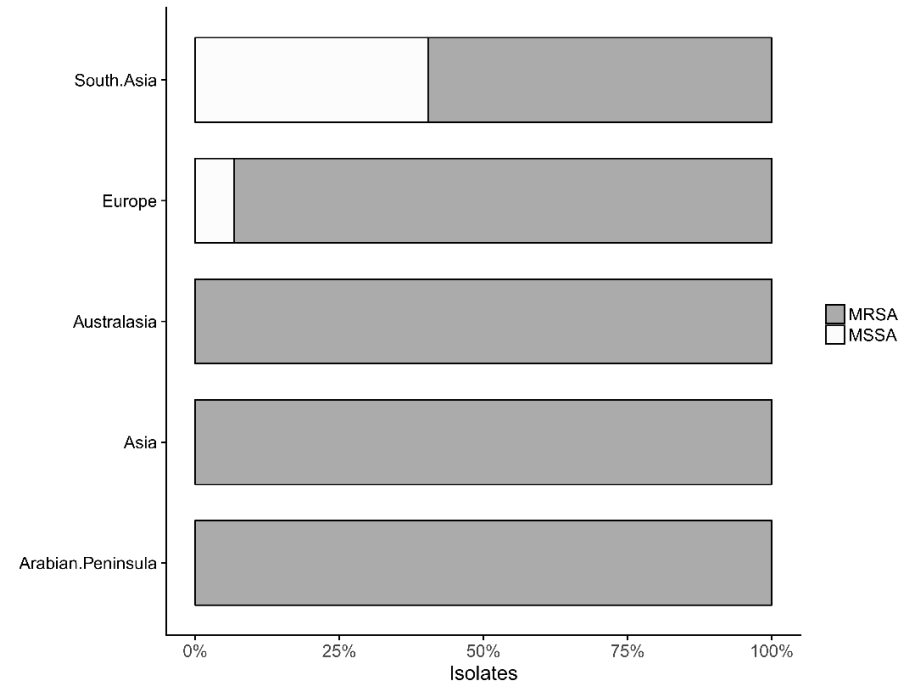

**D**

**a**

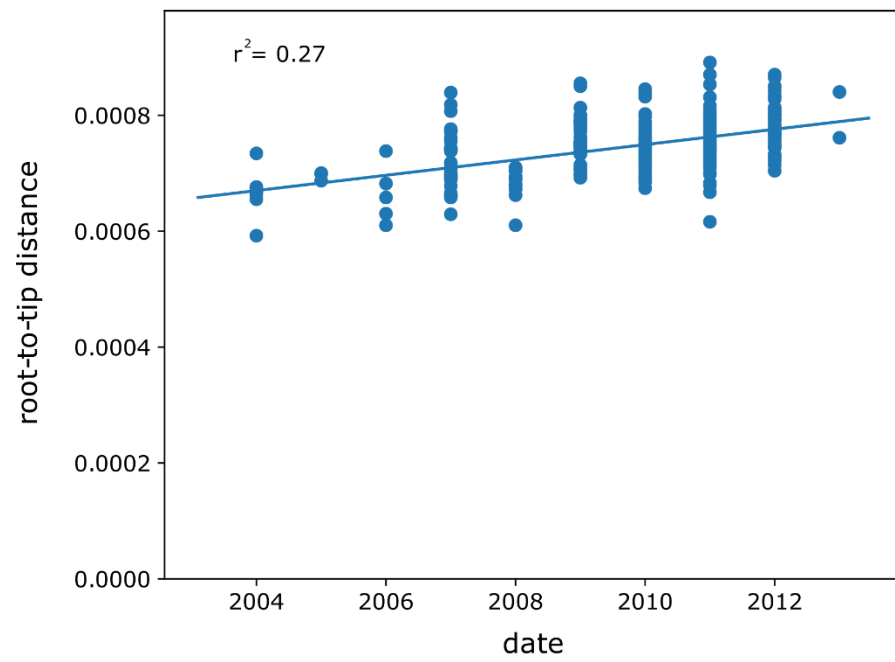

**b**

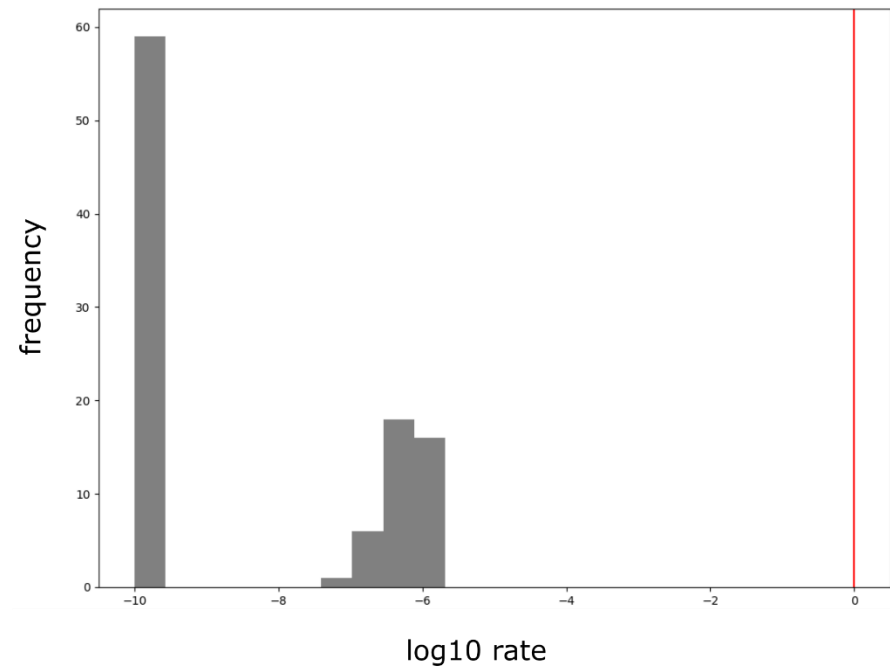

Supplement: FIG S1 [file mBio.01105-19-sf001.pdf]

# Epidemiology of ST772

- South Asia
  - Outside South Asia
  - Unknown
- 1st Ring: Family  
2nd Ring: Travel Nodes

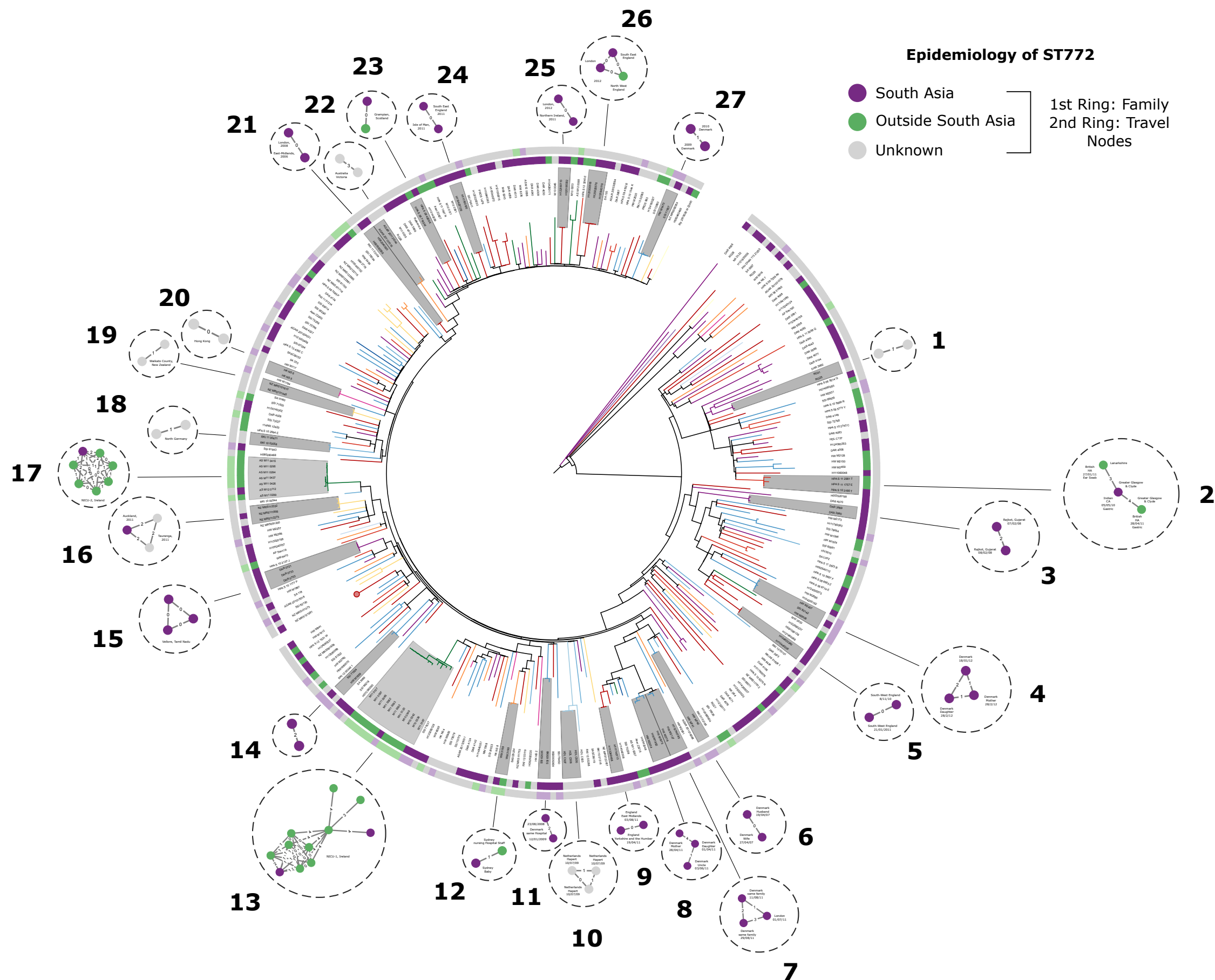

Supplement: FIG S2 [file mBio.01105-19-sf002.pdf]

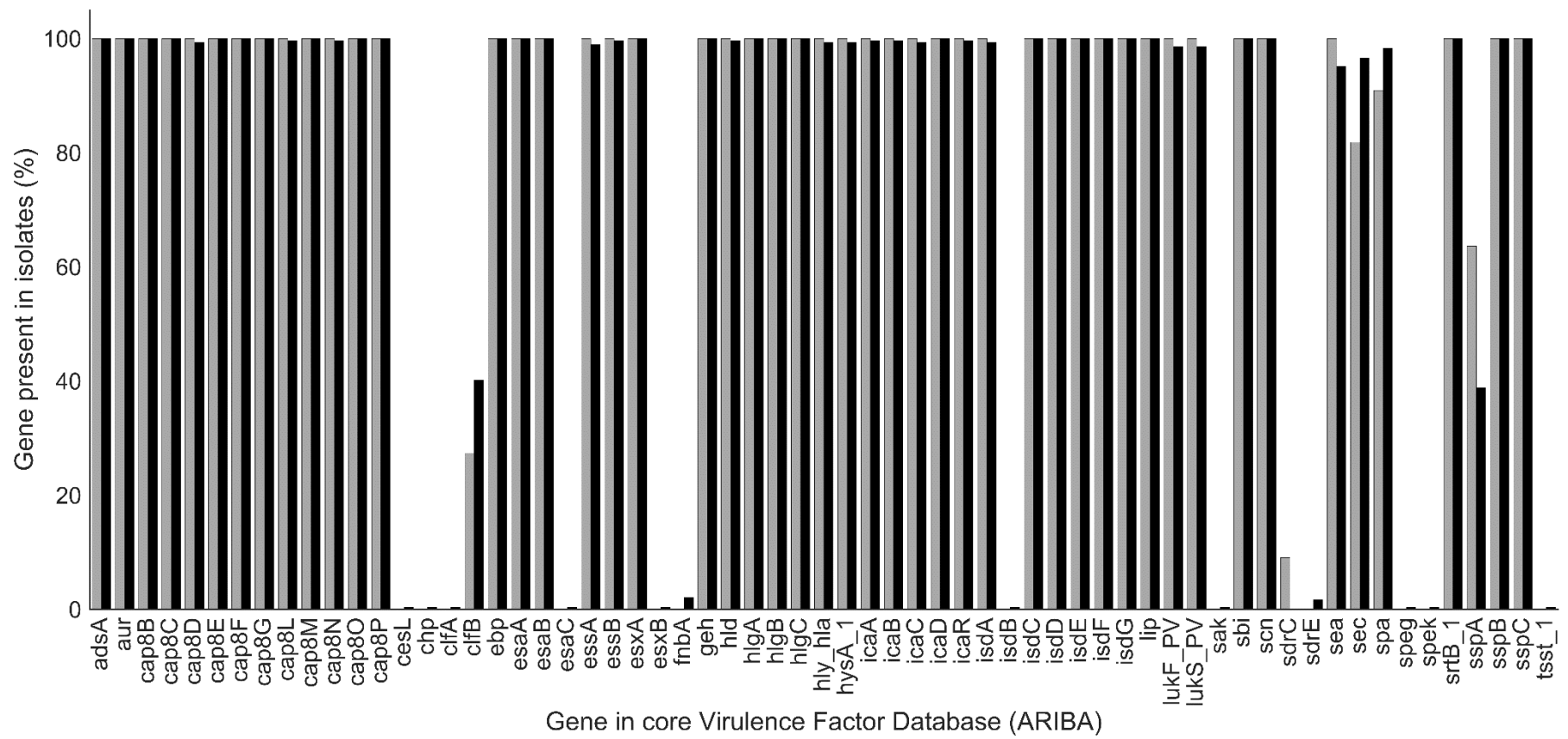

Supplement: FIG S3 [file mBio.01105-19-sf003.pdf]

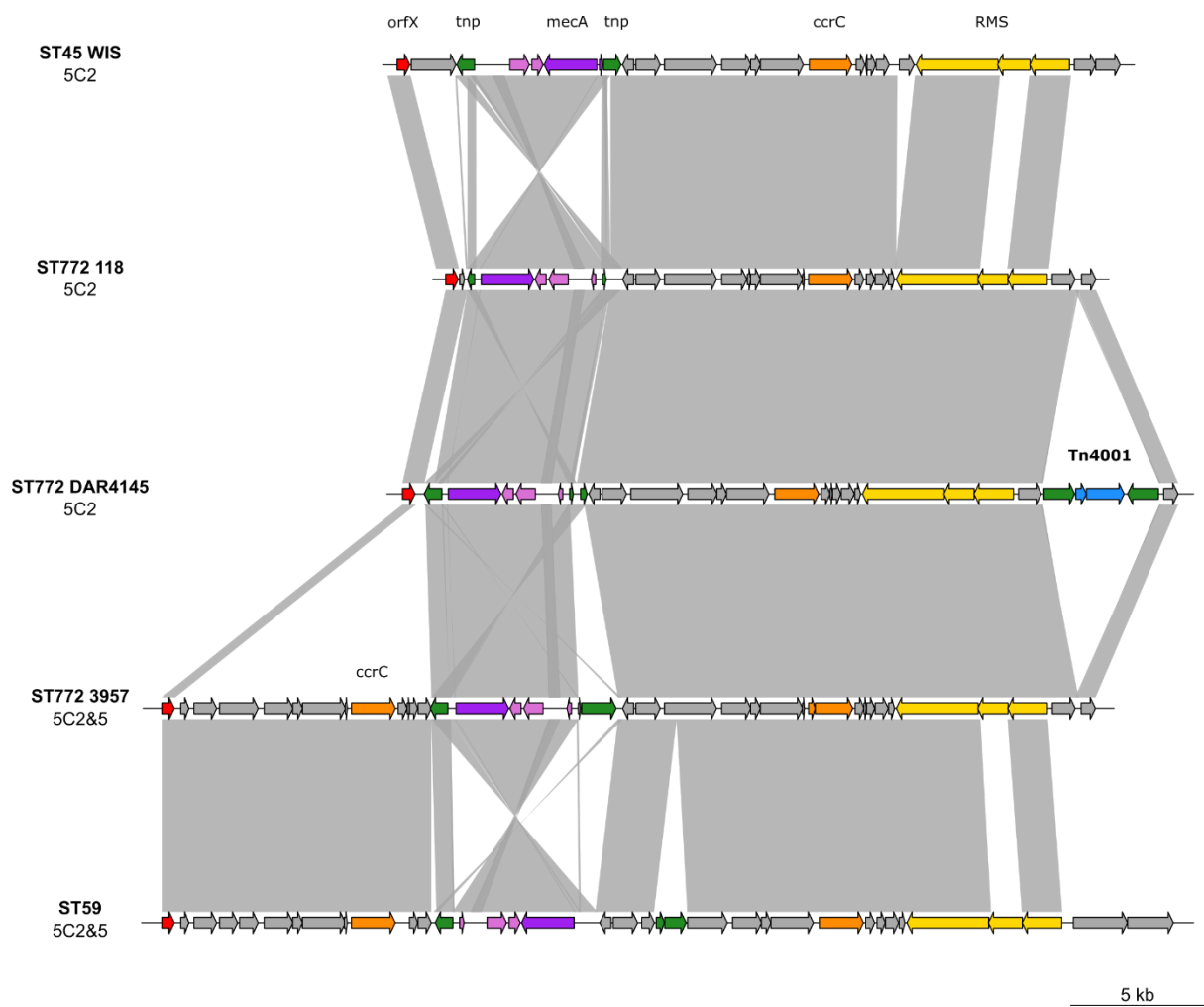

Supplement: FIG S4 [file mBio.01105-19-sf004.pdf]

**a**

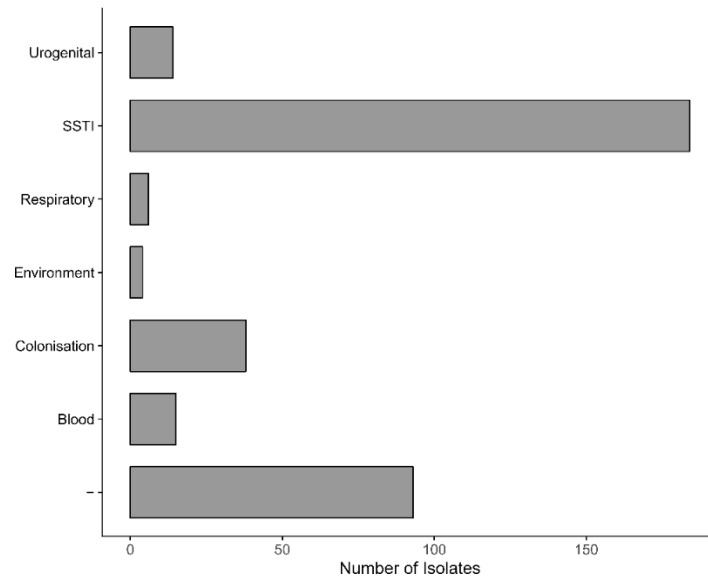

**b**

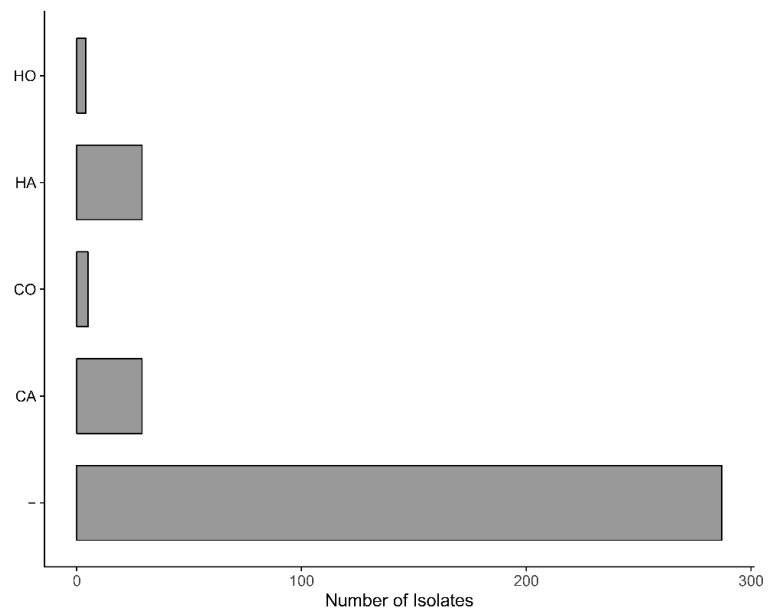

Supplement: FIG S5 [file mBio.01105-19-sf005.pdf]

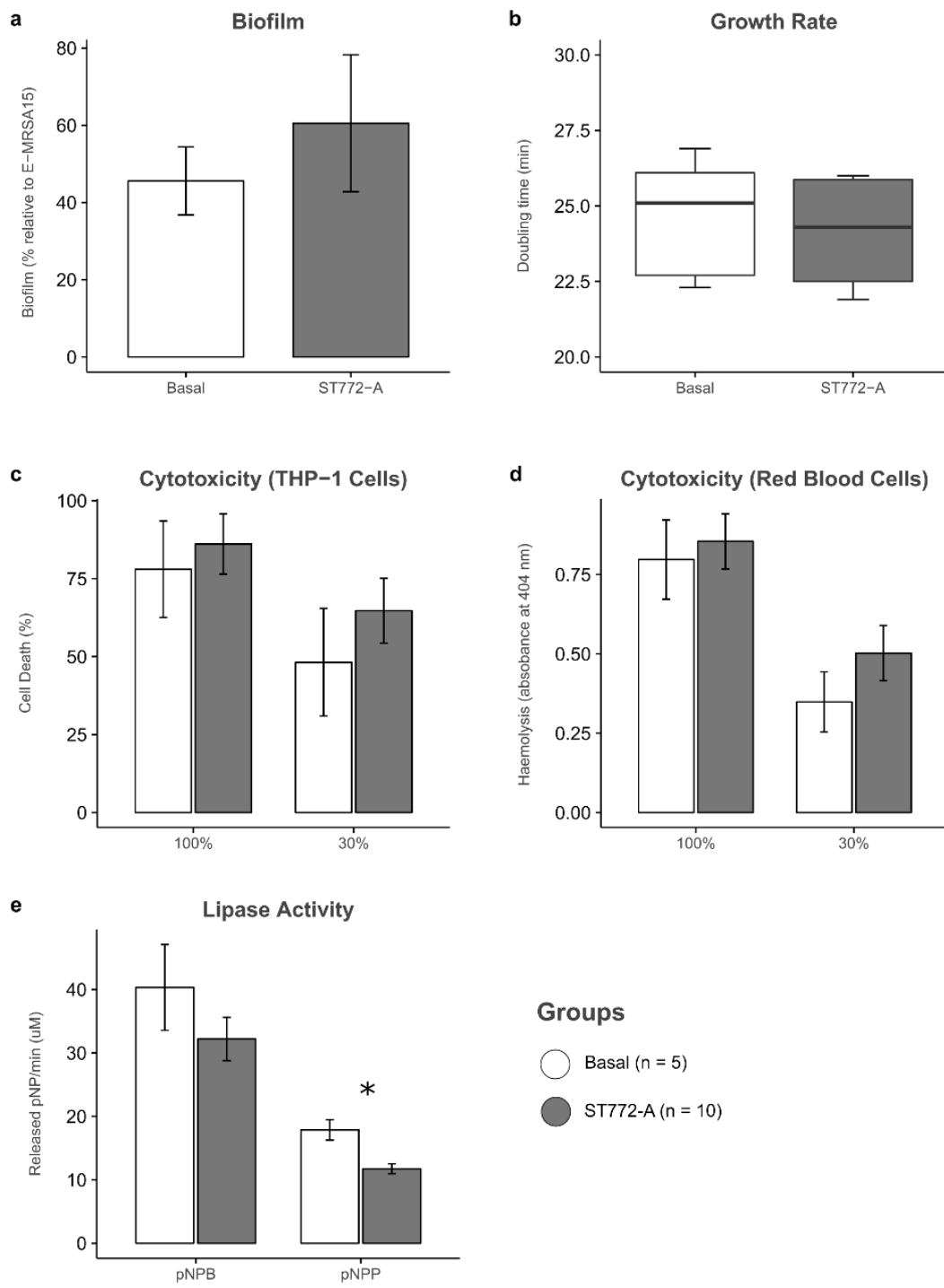

Supplement: FIG S6 [file mBio.01105-19-sf006.pdf]

A

a

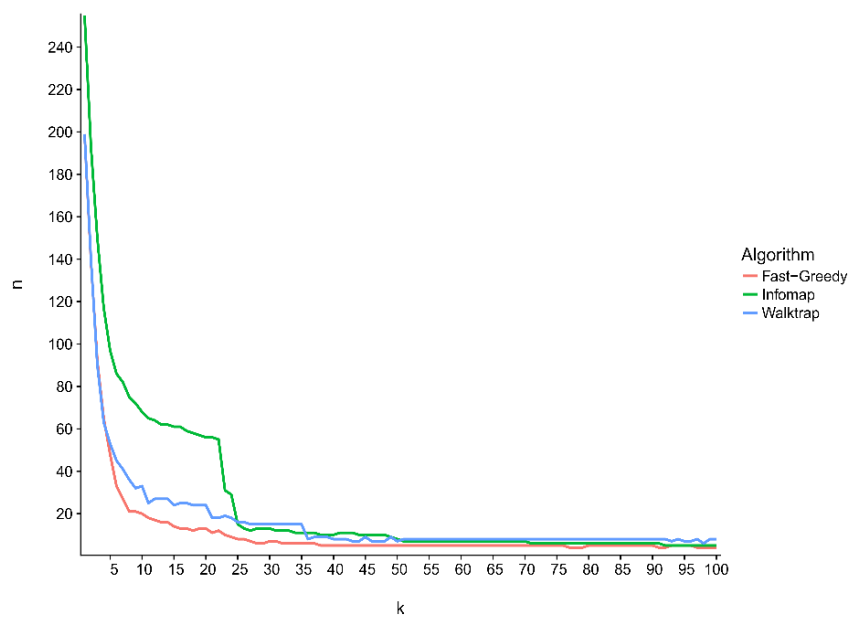

b

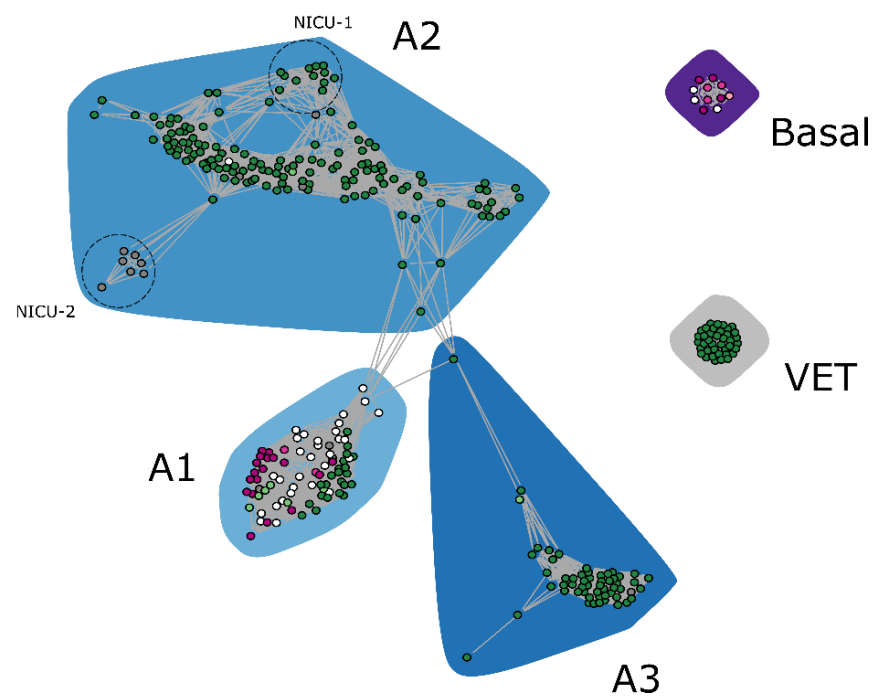

# B

## a

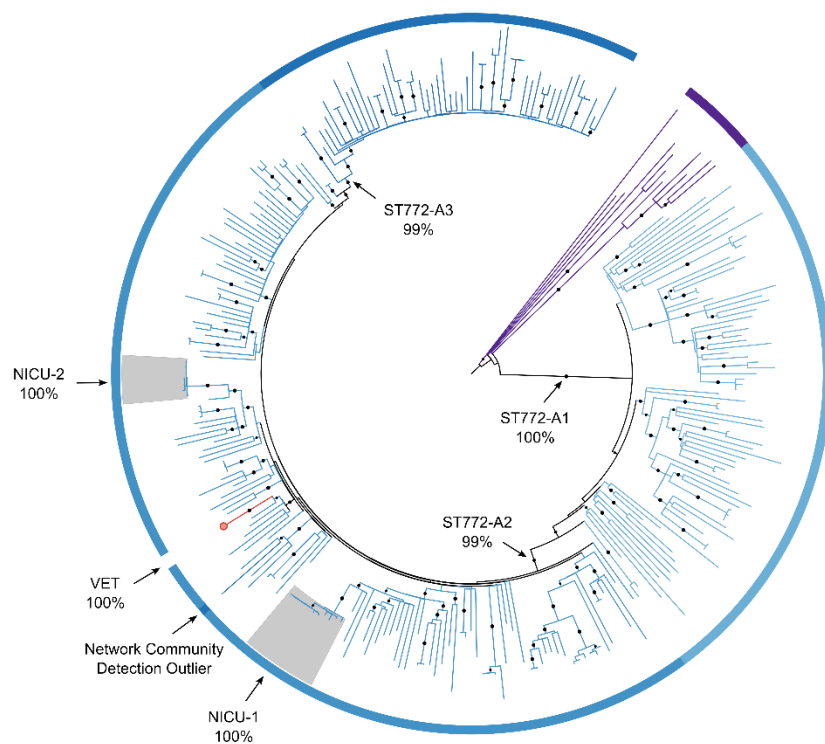

## b

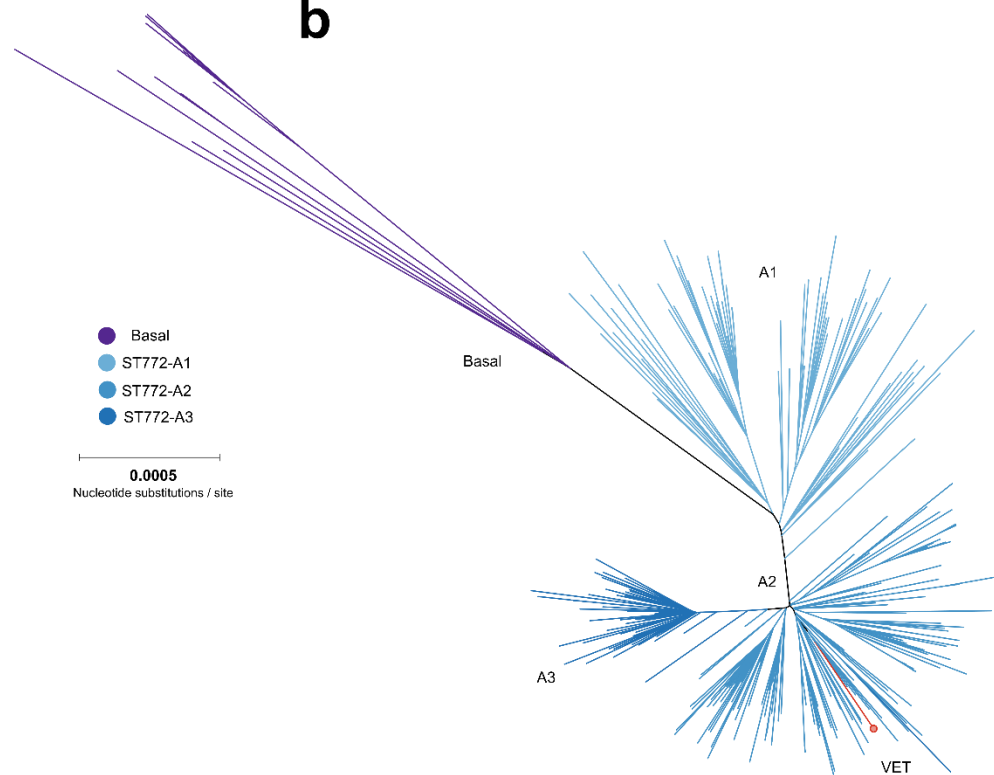

Supplement: FIG S7 [file mBio.01105-19-sf007.pdf]

**a**

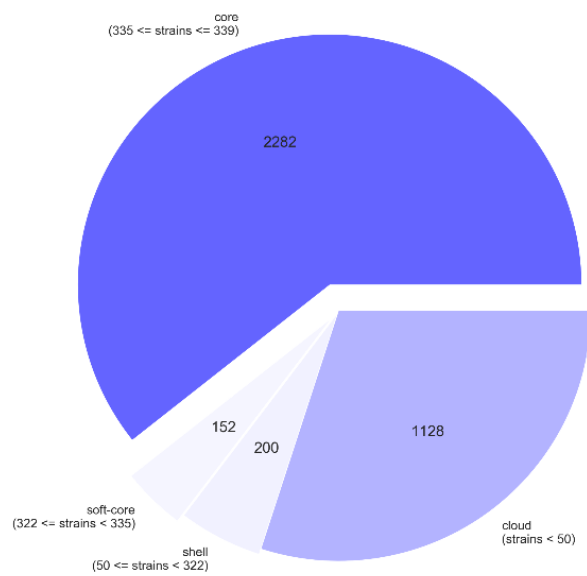

**b**

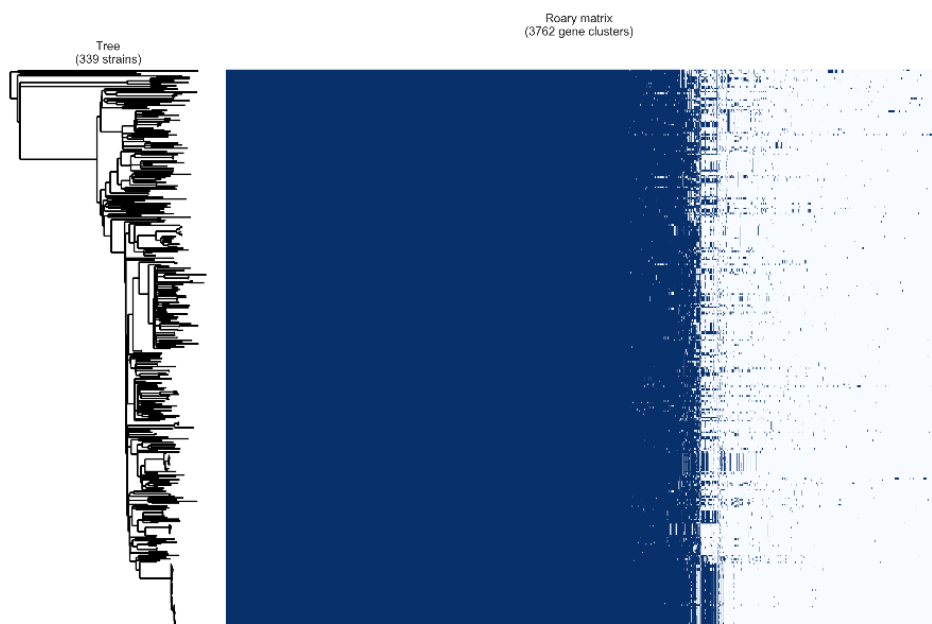

Supplement: FIG S8 [file mBio.01105-19-sf008.pdf]
